# Thiophene-Based Optical Ligands That Selectively Detect Aβ Pathology in Alzheimer’s Disease

**DOI:** 10.1002/cbic.202100199

**Published:** 2021-06-21

**Authors:** Therése Klingstedt, Hamid Shirani, Bernardino Ghetti, Ruben Vidal, K. Peter R. Nilsson

**Affiliations:** [a] Department of Physics, Chemistry and Biology Linköping University, SE-581 83 Linköping (Sweden); [b] Department of Pathology and Laboratory Medicine Indiana University School of Medicine Indianapolis, 46202, Indiana (USA)

**Keywords:** Alzheimer’s disease, amyloid beta-peptides, fluorescence, protein aggregates, tau

## Abstract

In several neurodegenerative diseases, the presence of aggregates of specific proteins in the brain is a significant pathological hallmark; thus, developing ligands able to bind to the aggregated proteins is essential for any effort related to imaging and therapeutics. Here we report the synthesis of thiophene-based ligands containing nitrogen heterocycles. The ligands selectively recognized amyloid-β (Aβ) aggregates in brain tissue from individuals diagnosed neuropathologically as having Alzheimer’s disease (AD). The selectivity for Aβ was dependent on the position of nitrogen in the heterocyclic compounds, and the ability to bind Aβ was shown to be reduced when introducing anionic substituents on the thiophene backbone. Our findings provide the structural and functional basis for the development of ligands that can differentiate between aggregated proteinaceous species comprised of distinct proteins. These ligands might also be powerful tools for studying the pathogenesis of Aβ aggregation and for designing molecules for imaging of Aβ pathology.

## Graphical Abstract

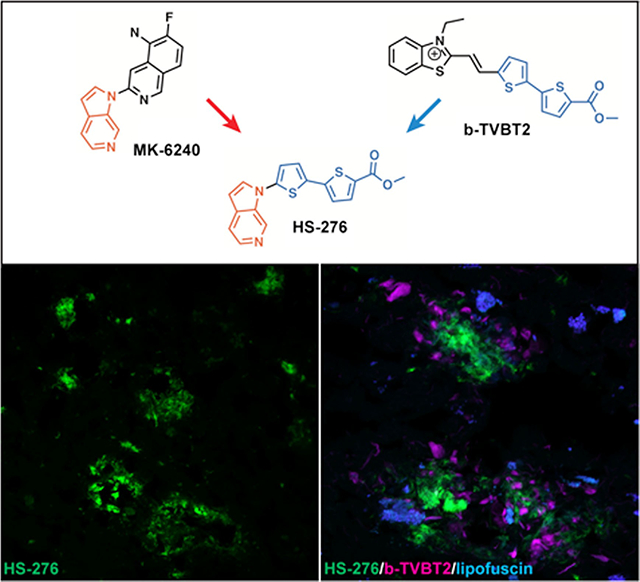

**Molecular matchmaking:** Ligands MK-6240 and b-TVBT2 are selective for aggregated tau in Alzheimer’s disease (AD). When combining the azaindole moiety of MK-6240 with the methyl-bithiophene-carboxylate unit of b-TVBT2, the resulting ligand, termed HS-276, does not show binding to tau but instead high selectivity for Aβ aggregates in AD brain tissue. By introducing small structural alterations of the HS-276 scaffold, the binding properties change.

## Introduction

Alzheimer’s disease (AD) is the most common neurodegenerative disease and the leading cause of dementia accounting for 50–70% of all cases.^[1]^ Similar to many other neurodegenerative diseases, AD is associated with the accumulation of protein aggregates in the brain. The histopathological hallmarks of AD are the presence of extracellular deposits composed by the amyloid-β (Aβ) peptide and intraneuronal neurofibrillary tangles (NFTs) made of hyperphosphorylated tau. The processes that cause the formation of Aβ plaques or NFTs are early events in the pathogenesis of the disease and begin occurring before the onset of clinical symptoms.^[2–6]^ Therefore, developing ligands that bind to these pathological lesions is the focus of many research groups. In fact, such ligands may aid in studying the pathogenesis of AD and enable early detection and longitudinal monitoring of pathological lesions.

The ^11^C-labelled benzothiazole Pittsburgh compound B (PIB) was the first ligand to be used clinically as a positron emission tomography (PET) tracer for Aβ aggregates and is still the gold standard for such purpose.^[7]^ Other Aβ selective ligands, such as florbetapir, florbetaben, flutemetamol and AZD4694, based on different heterocyclic molecular scaffolds, have also been developed and evaluated in clinical studies.^[8–11]^ For selective detection of tau filamentous aggregates, several classes of potential ligands have been identified. The first generation of ligands, which includes, for example, ^18^F-THK5351, ^18^F-AV-1451 (^18^F-T-807) and ^11^C-PBB3, has shown encouraging results, but unexplained retention of ligands in several brain regions has been reported.^[12–16]^ Therefore, a second-generation of ligands for tau imaging is now being developed with the aim of reducing the previously observed off-target binding. Structurally, the new ligands are either modified versions of first-generation tracers, as for example ^18^F-PI-2620, or are designed by using novel chemical scaffolds, as in the case of ^18^F-MK-6240.^[17,18]^

Even though a variety of ligands for detection of Aβ or tau aggregates now exist, there have been examples where they have failed to label their respective targets.^[19–22]^ The lack of detection of certain aggregated species of proteins might be caused by the existence of distinct morphotypes of Aβ or tau aggregates. The prion protein is a classic example of how an identical protein primary sequence can form distinct aggregate morphotypes giving rise to specific neuropathological and clinical abnormalities (i.e. prion strains).^[23]^ A similar polymorphism has been suggested both for Aβ^[24–30]^ and tau aggregates.^[31–34]^ For example, by using cryo-electron microscopy (cryo-EM), Aβ fibrils derived from AD brain were shown to be polymorphic.^[35]^ Recent cryo-EM studies have also revealed distinct structures of tau filaments purified from the brains of patients diagnosed with AD, chronic traumatic encephalopathy, corticobasal degeneration or Pick’s disease, as well as filaments composed of recombinant tau.^[36–40]^

**Figure 1.**
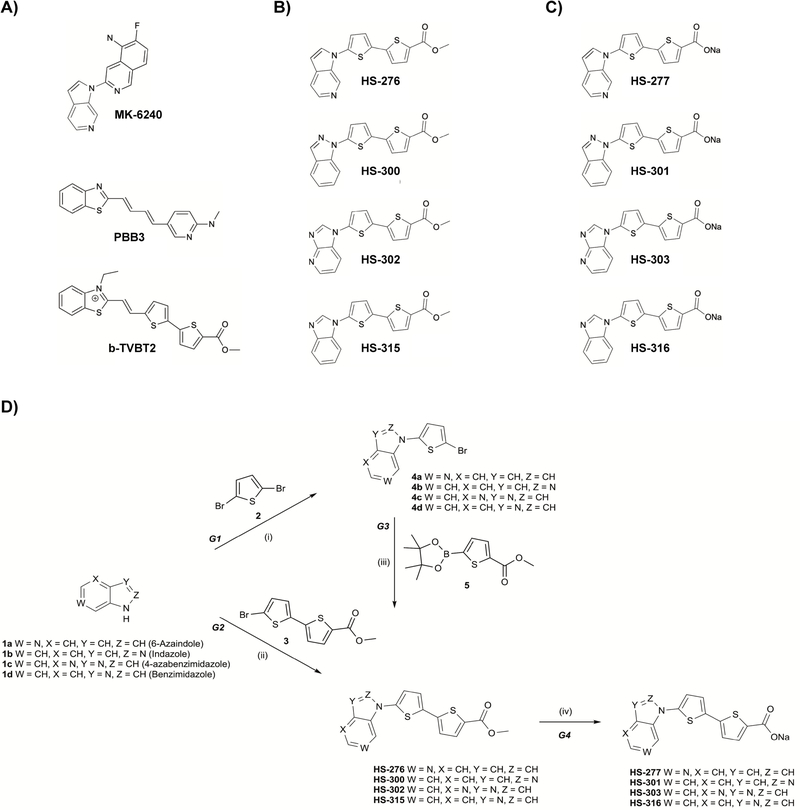
Chemical structures and synthesis of thiophene-based ligands mimicking MK-6240. **A)** Chemical structures of previously reported ligands, MK-6240 (top), PBB3 (middle) and b-TVBT2 (bottom) that can be utilized for imaging of tau aggregates. **B**,**C)** Chemical structures of novel thiophene-based ligands mimicking MK-6240. **D)** Reagents and conditions: (i) (***G1***) DMF, CuI, Cs_2_CO_3_, 180°C, 30 min, microwave; (ii) (***G2***) Toluene, CuI, *N,N’*-dimethylenediamine, K_3_PO_4_, 110°C, 24 h; (iii) (***G3***) 1,4-dioxane/MeOH, PEPPSI™-IPr, K_2_CO_3_, 80°C, 1–3 h; (iv) (***G4***) a) LiOH (3M), 1,4-dioxane, 80°C; b) HCl, 1,4-dioxane/H_2_O, NaOH (1 M).

Lately, a novel class of optical ligands known as conjugated poly- or oligothiophenes (LCPs and LCOs) has been used to study conformational variation of aggregates composed of proteins and peptides such as the prion protein, Aβ, tau and α-synuclein.^[41–48]^ In addition, the possibility of designing thiophene-based ligands that selectively target Aβ or tau aggregates in AD was recently demonstrated.^[49,50]^ For tau, bithiophene-vinyl-benzothiazole (b-TVBT) ligands were synthesized by replacing the pyridinyl-butadienyl-motif structure of tracer PBB3 (Figure 1A) with a bi-thiophenevinyl moiety.^[50,51]^ When evaluated on AD brain tissue sections, the b-TVBTs showed binding to NFTs, dystrophic neurites and neuropil threads (NTs), and their affinity for tau was higher as compared to PBB3.^[50]^ Hence, by introducing thiophene moieties to modify the structure of PBB3, the binding potential for tau appeared to increase. Herein, we have synthesized a library of ligands using the second-generation tau tracer MK-6240^[18]^ (Figure 1A) as a template. The overall aim of the study was to explore the effect of combining thiophene-based ligands with the MK-6240 scaffold in regard to binding to Aβ and tau aggregates in AD. By replacing the 5-amino-isoquinoline motif of MK-6240 with bithiophene units and linking them with azaindole or similar nitrogen containing heterocyclic compounds, eight structurally diverse ligands could be designed. When applying these ligands on AD brain tissue sections, the result clearly showed that the overall charge of the bithiophene unit, as well as the position of nitrogen in the heterocyclic compounds, have great impact on ligand binding to aggregates of Aβ or tau.

## Results

### Synthesis and optical characterization of thiophene-based ligands mimicking MK-6240

MK-6240 contains two molecular motifs, an azaindole and a 5-amino-isoquinoline moiety (Figure 1A). To generate a small library of thiophene-based ligands resembling MK-2640, the 5-amino-isoquinoline motif was first replaced by a methyl-bithiophene-carboxylate building block to render ligand HS-276 (Figure 1B). Secondly, three ligands, HS-300, HS-302 and HS-315 (Figure 1B) were created, that have the same methyl-bithiophene-carboxylate component as HS-276, but the azaindole unit respectively replaced by an indazole (HS-300), an azabenzimidazole (HS-302) or a benzimidazole (HS-315) building block. All these ligands were afforded in moderately good yields using two different synthetic routes (Figure 1D). Initially, the ligands were synthesized according to Buchwald arylation methodology^[52]^ involving copper (I) catalysed cross-coupling between the nitrogen heterocycles **1a**–**1d** and methyl-bithiophene-carboxylate (3). Although the arylation of 6-azaindole **1a** and indazole **1b** gave the target molecules HS-276 and HS-300 in good yields, this condition provided incomplete conversion of the starting material when applied to imidazoles **1c** and **1d** (typically 5 to 10 percent). Change of reaction conditions such as the reaction time, solvent or various kinds of bases and ligands had very little effect on the improvement of the product’s overall yields. Therefore, we evaluated the scope of arylation with slight modifications of the Buchwald method under ligand-free conditions towards intermediates **4a**–**4d** in a microwave system. A Suzuki–Miyaura cross-coupling reaction between these intermediates and pinacolboronic reagent (5) provided the corresponding products with a relatively better overall yield. Finally, four different analogues, HS-277, HS-301, HS-303 and HS-316 (Figure 1C), having an anionic bithiophene-carboxylate building block attached to the corresponding nitrogen containing heterocyclic element discussed above, were afforded by removing the methyl group from HS-276, HS-300, HS-302 and HS-315 (Figure 1D).

When dissolved in dimethyl sulfoxide (DMSO) or water, all ligands displayed distinctive absorption characteristics between 300 nm to 425 nm (Figure S1). HS-276 showed an absorption maximum at 370 nm, whereas the other ligands, with the azaindole unit replaced by an indazole (HS-300), an azabenzimidazole (HS-302) or a benzimidazole (HS-315) building block, revealed red-shifted (HS-300) or blue-shifted (HS-302 and HS-315) absorption maxima. Upon excitation with the respective absorption maximum, HS-276 and HS-300 showed similar spectra with an emission maximum around 480 nm, whereas HS-302 and HS-315 displayed emission spectra with two emission maxima at 450 and 470 nm (Figure S1). Thus, the position of heterocyclic nitrogen influenced the optical characteristics of the ligand. In addition, all the anionic ligands demonstrated a slight blue-shift of the absorption maximum, as well as a more pronounced blue-shift of the emission, compared to their uncharged counterpart (Figure S1).

### Substitution of benzothiazole with azaindole results in a shift of selectivity from tau to Aβ

**Figure 2.**
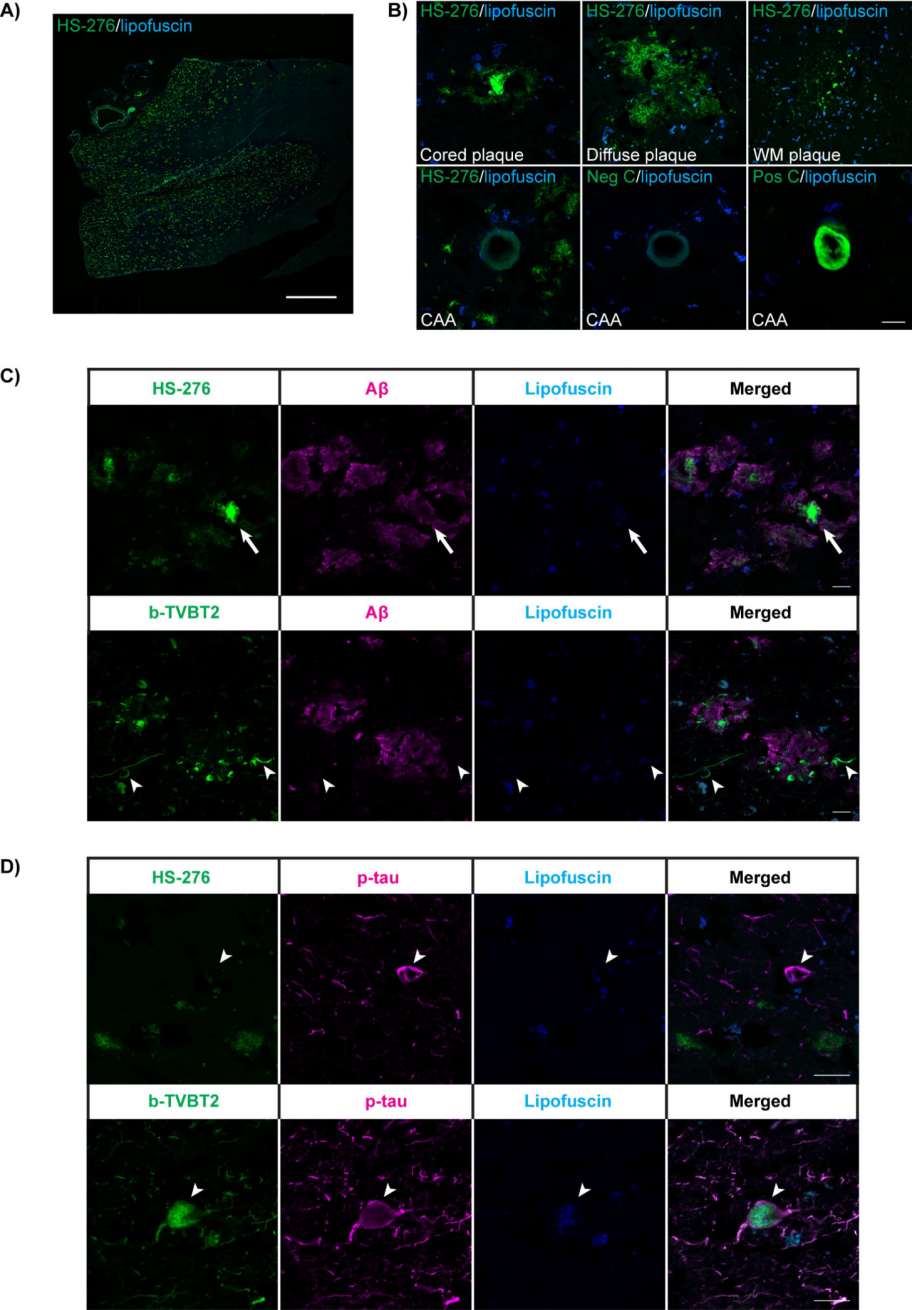
Substitution of benzothiazole with azaindole results in a shift of selectivity from tau to Aβ. **A)** Fluorescence image of AD brain tissue section showing the staining pattern of ligand HS-276 in green. The blue structures represent autofluorescent lipofuscin. Scale bar, 2 mm. **B)** Fluorescence images depicting different types of Aβ deposits in AD brain tissue section labelled with HS-276 (green) including cored plaque (left, top panel), diffuse plaque (middle, top panel) and white matter plaque (WM, right, top panel). In the bottom panel, the staining result of a cerebral amyloid angiopathy (CAA) lesion in AD brain tissue section is shown. The positive control (Pos C, right) represents staining with LCO ligand HS-84, whereas the negative control section (Neg C, middle) was incubated with PBS buffer only. In the section stained with HS-276, the fluorescence from the CAA lesion is similar to that observed in Neg C, indicating that HS-276 does not label vascular Aβ deposits. Autofluorescence from lipofuscin can be seen in blue. Scale bar, 20 μm. **C)** Fluorescence images of AD brain tissue sections labelled with anti-Aβ-antibody (magenta, 6E10) and ligand HS-276 (green, top panel) or b-TVBT2 (green, bottom panel). In the structure of HS-276, instead of the benzothiazole moiety found in b-TVBT2, the bithiophene unit is linked to an azaindole. The substitution resulted in a shift from tau to Aβ selectivity as HS-276 demonstrated binding to Aβ plaques and co-staining with the antibody (arrow), whereas b-TVBT2 only labelled structures resembling tau pathology (arrowhead). Note that the strong fluorescence from HS-276 when binding to the core of cored plaques dictated the intensity of the excitation light when acquiring the image. This makes it harder to distinguish the surrounding halo of the cored plaques and also other plaque types due to weaker fluorescence emitted from these structures. In the image, autofluorescence from lipofuscin is also shown (blue). Scale bar, 20 μm. **D)** Fluorescence images of AD brain tissue sections labelled with anti-phospho-tau (p-tau) antibody (magenta, AT8) and ligand HS-276 (green, top panel) or b-TVBT2 (green, bottom panel). HS-276 did not show any co-localization with the antibody (arrowhead), whereas immuno-positive tau aggregates, such as NFTs, were readily labelled by b-TVBT2 (arrowhead). Autofluorescent lipofuscin is shown in blue. Scale bar, 20 μm.

The first step in the biological characterization of the new ligand family was that of comparing, in neuropathologically diagnosed AD brain tissue, their binding properties with those recently demonstrated using the tau selective b-TVBT ligands.^[50]^ For this experiment, we chose to include HS-276 and b-TVBT2 (Figure 1A) as they share the same methyl-bithiophene-carboxylate building block in their chemical structure; however, in place of the benzothiazole moiety found in b-TVBT2, HS-276 contains an azaindole (Figure 1A, B). When applied on an AD brain section, the staining pattern of HS-276 was different compared to what was recently reported for b-TVBT2.^[50]^ Instead of binding to tau pathology, the ligand was labelling Aβ accumulations, including cored plaques and diffuse plaques in the grey matter as well as deposits in the white matter (Figure 2A, B). The fluorescence intensity of HS-276 upon binding was strong for all plaque types and they could easily be detected; however, the core of cored plaques exhibited much higher intensity of HS-276 emission compared to the diffuse plaques or the halo surrounding the core of cored plaques (Figure 2B). The cerebral amyloid angiopathy (CAA) lesions present in the tissue, confirmed by staining with LCO ligand HS-84, which has earlier been used for this purpose,^[53]^ were not labelled by HS-276 (Figure 2B). By combining HS-276 or b-TVBT2 with antibodies against Aβ (6E10) or hyperphosphorylated tau (AT8) on AD brain sections, the results clearly showed, once again, that the ligands were labelling different structures. HS-276 was binding to the various types of immuno-labelled Aβ accumulations, whereas, as expected, b-TVBT2 and the anti-Aβ antibody did not co-localize (Figure 2C). The emission maximum of HS-276 upon interacting with Aβ was 460 nm (Figure S2). The corresponding value for HS-276 when diluted in PBS buffer only was at 496 nm (Figure S2A). The blue-shift in emission indicates that binding to Aβ aggregates locks the ligand in a different conformation compared to when it is free in solution. When tissue sections were incubated with the ligand and the anti-tau antibody AT8, it was confirmed that HS-276 did not bind to tau immuno-positive pathologies, whereas b-TVBT2, as previously reported, labelled hyperphosphorylated tau (Figure 2D). Hence, in this comparison of HS-276 and b-TVBT2 binding properties, the staining experiment showed that i) combining the methyl-bithiophene-carboxylate building block with azaindole instead of benzothiazole resulted in a shift from tau to Aβ selectivity, and ii) when substituting the 5-aminoisoquinoline motif of the tau tracer MK-6240 with a methyl-bithiophene-carboxylate building block, the resulting ligand, HS-276, did not show selective binding to tau in AD as MK-6240 but instead showed a high selectivity for Aβ aggregates.

In the brain tissue, autofluorescent lipofuscin granules were also observed. The granules are mainly composed of protein and lipid degradation residues of lysosomal digestion.^[54]^ Since lipofuscin has a broad autofluorescence,^[55]^ it was sometimes, depending on the used excitation intensity, observed in the same fluorescence channels as the ligands. Therefore, to confirm that these structures represented autofluorescence and not ligand binding, an additional channel, in which the acquisition settings only allowed lipofuscin excitation, was used.

### Combining ligands to distinguish between Aβ and tau pathology in AD brain tissue

**Figure 3.**
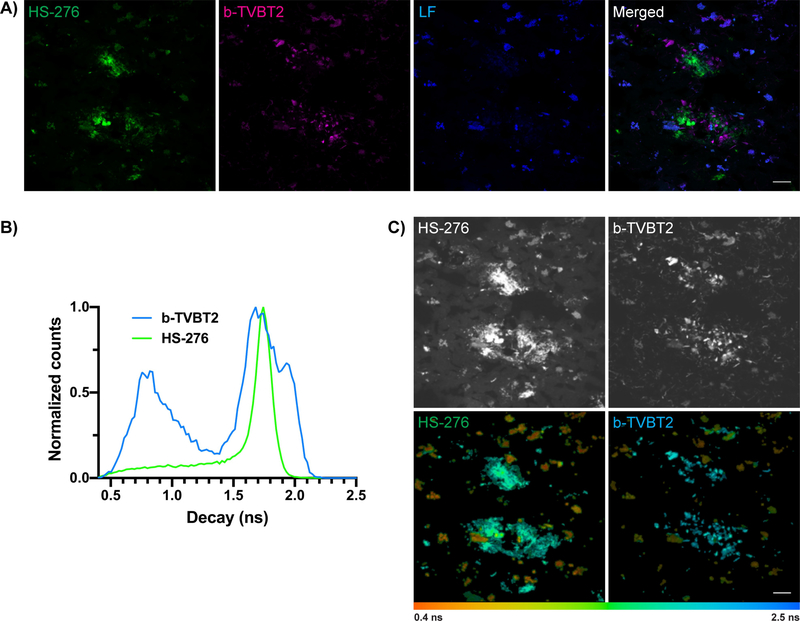
Combining ligands to distinguish between Aβ and tau pathology in AD brain tissue. **A)** Fluorescence image of AD brain section stained with HS-276 and b-TVBT2 simultaneously. The emission from HS-276 (green) and b-TVBT2 (magenta) could be separated into two channels, which allowed differentiation of Aβ and tau pathology based on the colour of the emitted light. Autofluorescence from lipofuscin granules is also displayed (blue). Scale bar, 20 μm. **B)** Intensity-weighted mean lifetime (ti) distributions of HS-276 labelled Aβ deposits (green) and b-TVBT2 labelled tau pathologies (blue) in the AD brain section. For the latter, the shorter lifetime distributions (0.5 ns to 1.2 ns) were observed due to autofluorescent lipofuscin. **C)** Fluorescence intensity (top) and fluorescence lifetime (bottom) images of HS-276 (left) and b-TVBT2 (right) positive pathologies in the double labelled AD brain section described in panel A. In the fluorescence lifetime images, the colour bar represents lifetimes from 0.4 ns (orange) to 2.5 ns (blue) and the images are colour coded according to lifetime. Scale bar, 20 μm.

After establishing that HS-276 was only binding to Aβ aggregates in AD tissue, we next wanted to explore if this ligand, in combination with the tau selective ligand b-TVBT2, could be used to distinguish between Aβ and tau pathologies. Since the emission maximum of HS-276 when binding to Aβ was at 460 nm (Figure S2A), and the corresponding value for b-TVBT2 emission when binding to NFTs has been reported to be 600 nm,^[50]^ it should be possible to separate Aβ and tau without any overlap of the emission. In addition, the two ligands can be excited with two different lasers, 405 nm (HS-276) and 580 nm (b-TVBT2). An AD brain tissue section was stained with a solution containing 100 nM HS-276 and 100 nM b-TVBT2, and Aβ and tau binding was assessed by measuring the emission from HS-276 and b-TVBT4 upon binding, using two different channels (Figure 3A). The result showed that the combination of ligands could be used to distinguish between Aβ and tau pathology in AD brain tissue. The fluorescent properties of the ligands when interacting with the aggregated proteins were also explored using fluorescence lifetime imaging microscopy (FLIM). By plotting the acquired data, the decay time of each ligand could be specified as 1.5 ns to 1.9 ns for HS-276 and 1.4 ns to 2.1 ns for b-TVBT2 (Figure 3B). Autofluorescent lipofuscin could also be seen; however, as the lifetime of lipofuscin was shorter (0.5 ns to 1.2 ns) than the values obtained for HS-276 and b-TVBT2, this structure could easily be distinguished from both Aβ and tau (Figure 3B, C). The generated FLIM images, in which the fluorescence decay times were visualized and color-coded accordingly, revealed that the lifetime distributions of HS-276 was similar to that of b-TVBT2 (Figure 3C).

### The position of heterocyclic nitrogen influences ligand binding to tau, but not to Aβ

**Figure 4.**
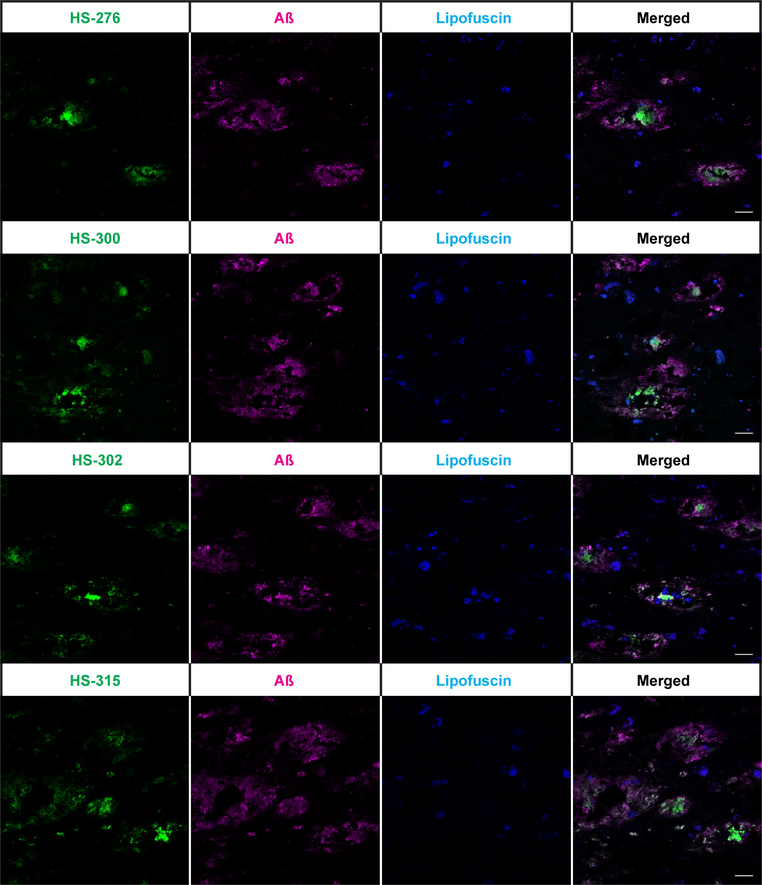
The position of heterocyclic nitrogen does not influence ligand binding to Aβ. Fluorescence images of brain tissue sections with AD pathology stained with the indicated ligand (green, see Figure 1 for ligand structures) and anti-Aβ antibody (magenta, 6E10). Autofluorescent lipofuscin is also show (blue). For each ligand, the arrangement of nitrogen in the heterocyclic aromatic compound differed. As all included ligands showed binding to Aβ plaques and co-labelling with the antibody, the result indicated that the position of the heterocyclic nitrogen did not have an effect on ligand binding to Aβ plaques. Scale bar, 20 μm.

We have previously reported that alterations of the heterocyclic aromatic ring have an impact on ligand binding to protein aggregates in AD.^[50]^ Therefore, next, we wanted to investigate if rearranging the nitrogen atoms in the heterocyclic unit of HS-276, while keeping the methyl-bithiophene-carboxylate component intact, would have any effect on ligand binding to Aβ plaques. As described earlier, three ligands were synthesised, in which the azaindole unit was replaced by an indazole (HS-300), an azabenzimidazole (HS-302) or a benzimidazole (HS-315) building block (Figure 1B). The ligands were then evaluated by performing ligand and antibody double staining on AD brain tissue sections. Similar to HS-276, all three ligands demonstrated co-staining with the Aβ antibody 6E10, which confirmed that also the newly introduced nitrogen heterocycles, in combination with the methyl-bithiophene-carboxylate moiety, resulted in binding to various types of Aβ plaques such as cored plaques and diffuse plaques (Figure 4).

**Figure 5.**
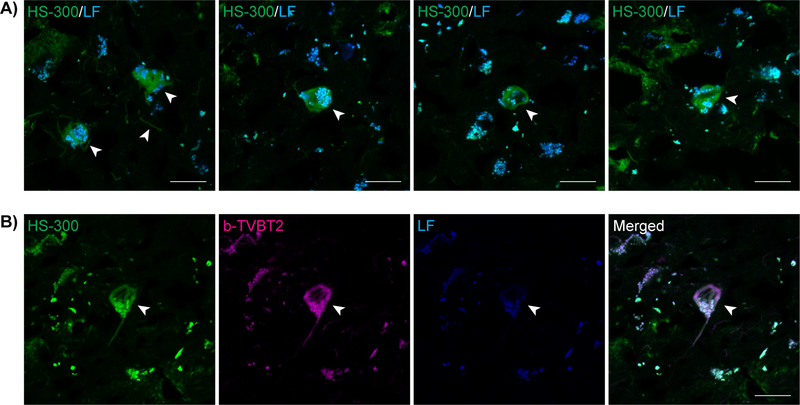
The position of heterocyclic nitrogen influences ligand binding to tau. **A)** Fluorescence images of AD brain tissue section labelled with ligand HS-300 (green). In addition to Aβ plaques, HS-300 was also binding to morphologies resembling NFTs and NTs (arrowhead). Blue/white structures are autofluorescent lipofuscin (LF) granules. Note, in all images, the intensity of the green and blue channel has been improved to enhance visualization. Scale bar, 20 μm. **B)** Fluorescence image of AD brain tissue section double-labelled with ligand HS-300 (green) and b-TVBT2 (magenta). HS-300 co-localized with b-TVBT2, confirming that the ligand was binding to tau inclusions. Autofluorescence from LF is depicted in blue. Note, the intensity of the green channel has been improved to enhance visualization. Scale bar, 20 μm.

The emission spectrum of each ligand upon binding was also collected, and the result showed that the emission maximum of HS-276, HS-300 and HS-301 was at 460 or 470 nm, whereas HS-302 and HS-315 emitted a more blue-shifted light with a maximum at 440 or 450 nm (Figure S2). Similar to HS-276, all ligands, except HS-301, showed a blue-shift in emission upon binding to Aβ aggregates compared to when being in buffer (Figure S2A). Interestingly, in addition to Aβ plaques, one of the ligands, HS-300, was also binding to morphologies resembling NFTs and NTs (Figure 5A). Since it is possible to distinguish between b-TVBT2 and HS-276 fluorescence (Figure 3A), and the emission maximum of HS-276 is similar to that of HS-300 (Figure S2), we performed a staining experiment in which HS-300 and b-TVBT2 were added to the same AD brain section. When analysing the result, it could be concluded that several structures were labelled with both ligands (Figure 5B). As the selectivity of b-TVBT2 for tau aggregates in AD has been established,^[50]^ the demonstration of co-staining with HS-300 and b-TVBT2 was a confirmation that the non-Aβ HS-300 positive structures were composed of tau aggregates. However, both when applied alone and in combination with b-TVBT2, the fluorescence intensity of HS-300 bound to tau pathologies was considerably weaker than its emission from Aβ plaques, and it was sometimes difficult to detect labelled tau inclusions. HS-300 staining was also performed in combination with AT8 tau antibody, but when the section was pre-stained with the antibody, the intensity of the light emitted from the ligand upon binding was very low (Figure S3) making it difficult to identify NFTs and NTs showing HS-300 and anti-tau double staining. A spectral comparison of HS-300 labelling Aβ or tau was also performed, and the result did not reveal any significant difference in emission (Figure S2C). In addition to HS-300, HS-302 and HS-315 also showed binding to tau; however, the number of labelled NFTs and NTs was much lower compared to what was observed with HS-300. In summary, by replacing the azaindole unit in HS-276 with other heterocyclic aromatic compounds, we observed that positional changes of the heterocyclic nitrogen did not have an effect on Aβ binding in AD brain tissue. It did, however, influence ligand labelling of tau aggregates, with the nitrogen arrangement found in indazole showing the highest affinity for tau.

### The charge of the ligand influences binding to Aβ and tau

**Figure 6.**
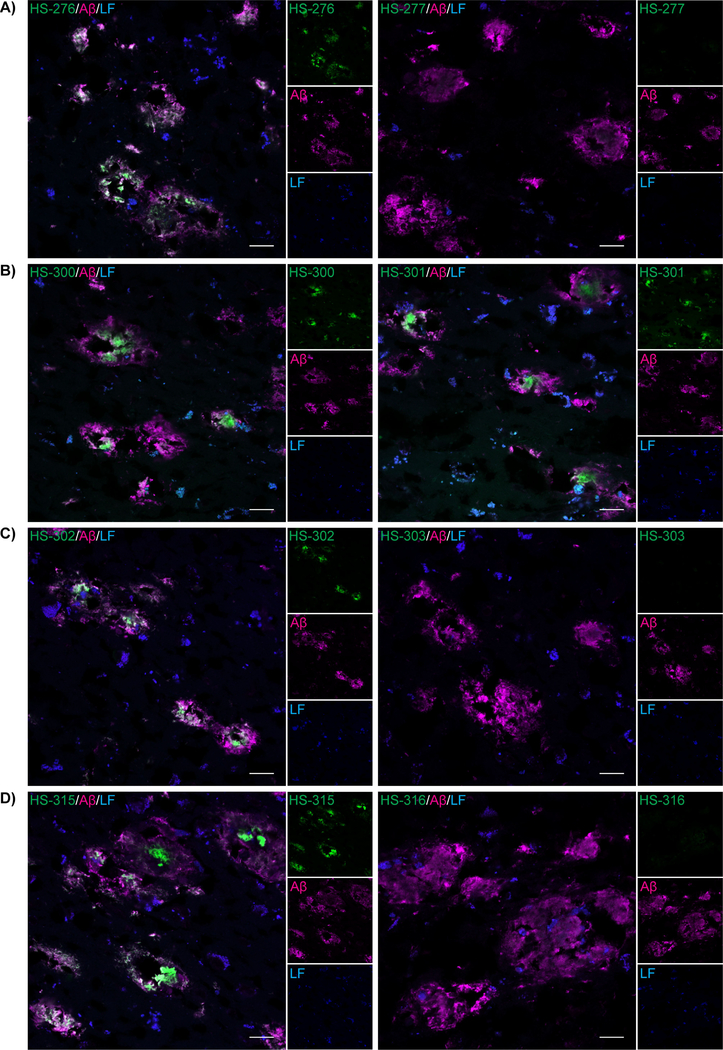
The charge of the ligand influences binding to Aβ and tau. **A**–**D)** Fluorescence images of brain tissue sections with AD pathology double-labelled with anti-Aβ-antibody (magenta, 6E10) and ligand HS-276 (A, left), HS-277 (A, right), HS-300 (B, left), HS-301 (B, right), HS-302 (C, left), HS-303 (C, right), HS-315 (D, left) or HS-316 (D, right). Ligand positivity is shown in green and autofluorescence from lipofuscin in blue. See Figure 1 for ligand chemical structures. All uncharged ligands, HS-276, HS-300, HS-302 and HS-315, showed binding to Aβ deposits and co-localization with the antibody. For HS-276 and HS-300, binding to Aβ plaques has already been confirmed (see Figure 2 and Figure 4), however, they were included in the experiment for comparison. Scale bar, 20 μm. **A**–**D)** When introducing an anionic substituent in the structure of the uncharged ligand HS-276, HS-302 or HS-315, the interaction with Aβ deposits was lost, and none of the resulting ligands, HS-277, HS-303 and HS-316, showed any positivity when applied to AD brain sections. **B)** The uncharged ligand HS-300 showed co-localisation with the Aβ antibody, and, in comparison to the other included uncharged ligand structures, introducing an anionic substituent in its structure did not have an effect on Aβ binding. The resulting ligand, HS-301, exhibited strong fluorescence upon its interaction with Aβ deposits and demonstrated co-localization with Aβ antibody.

The ligands evaluated so far, HS-276, HS-300, HS-302 and HS-315, all have a chemical structure that is uncharged (Figure 1B). The absolute majority of thiophene-based ligands developed in our laboratory, with the purpose of detecting and characterizing protein aggregates, has been anionic. Therefore, we next wanted to explore if the introduction of a negative charge on the thiophene-based ligands mimicking MK-6240 would have any effect on ligand binding to Aβ and/or tau aggregates in AD. As mentioned above, a set of four new anionic ligands were achieved by removal of the methyl group from HS-276, HS-300, HS-302 and HS-315. Staining with these anionic ligands, HS-277, HS-301, HS-303 and HS-316 (Figure 1C) in combination with an Aβ antibody on AD brain tissue sections revealed that none of the newly introduced ligands, with the exception of HS-301, showed any detectable binding to Aβ plaques or co-labelling with the antibody (Figure 6). In all samples, a weak and blue-shifted fluorescence could be seen from some of the plaques; however, as this emission was also evident when omitting the ligands (Figure S4), the observation was concluded as autofluorescence, which has been reported for Aβ plaques.^[56,57]^ As previously indicated, the only negatively charged ligand able to bind to Aβ was HS-301. The labelling pattern displayed by HS-301 overlapped with the antibody, and the interaction with the aggregated Aβ resulted in strong emission from the ligand (Figure 6). Structures morphologically resembling NFTs, NTs or dystrophic neurites could not be detected with HS-277, HS-303 or HS-316, while a few weakly positive NTs could be seen in sections stained with HS-301. Hence, introducing negatively charged groups in the bithiophene building block resulted in loss of binding to Aβ, except when the thiophene moiety was linked to an indazole unit (HS-301). Ligand interaction with tau was also inhibited when the negative charge was added to all of the molecular scaffolds.

### The ligands compete for binding of the anionic oligothiophene q-FTAA-CN to Aβ plaques

To investigate the binding mode of the new ligand scaffold to Aβ aggregates we performed a staining experiment in which AD brain tissue sections were pre-incubated with 10 μM of q-FTAA-CN or HS-169, two LCOs that have been reported as excellent tools for detection of Aβ deposits.^[49,53,58]^ After a washing step to remove unbound LCOs, the sections were stained with 100 nM HS-276.

**Figure 7.**
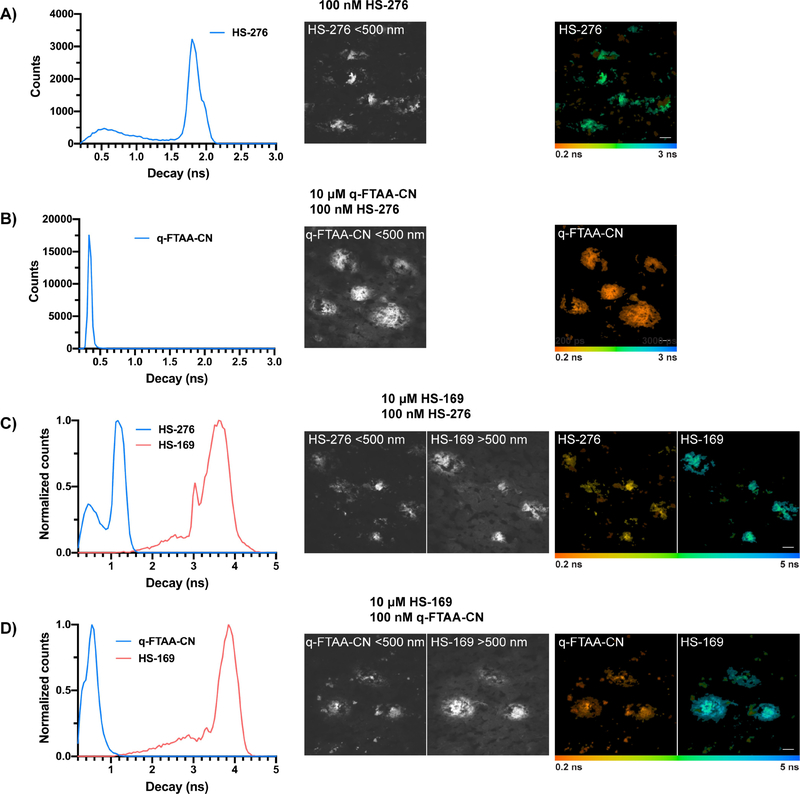
The ligands compete for binding of the anionic oligothiophene q-FTAA-CN to Aβ plaques. **A**–**D)** intensity-weighted mean lifetime (t_i_) distributions of (A) HS-276 (blue), (B) q-FTAA-CN (blue), (C) HS-276 (blue)/HS-169 (coral), or (D) q-FTAA-CN (blue)/HS-169 (coral) when binding to Aβ plaques in AD brain tissue sections (left panel). The middle and right panel, respectively, show fluorescence intensity and fluorescence lifetime images of AD brain tissue section incubated with (A) 100 nM HS-276, (B) 10 μM q-FTAA-CN and then 100 nM HS-276, (C) 10 μM HS-169 and then 100 nM HS-276, or (D) 10 μM HS-169 and then 100 nM q-FTAA-CN. PMT detectors registering photons with wavelengths below 500 nm (*<*500) or above 500 nm (*>*500) were used. In the fluorescence lifetime images the lifetimes are colour-coded according to the colour-bar representing lifetimes from 0.2–3 ns (A,B) or 0.2–5 ns (C,D). According to the results, pre-treatment with 10 μM q-FTAA-CN, but not 10 μM HS-169, blocked subsequent binding with HS-276 suggesting that q-FTAA-CN and HS-276 share analogous binding site on Aβ plaques. Scale bar, 20 μm.

The 100-fold excess of q-FTAA-CN or HS-169 made it difficult to spectrally identify each ligand as the intensity of the fluorescence emitted from HS-276, when exciting at 405 nm, was considerably lower than the emission from q-FTAA-CN or HS-169. Therefore, the possibility of detecting ligand binding by using FLIM was explored. Aβ deposits in a brain section that was only stained with 100 nM HS-276 displayed decay times between 1.6 ns to 2.1 ns (Figure 7A), which was similar to what was observed from HS-276 stained Aβ deposits in the HS-276 and b-TVBT2 double-stained tissue section (Figure 3B). In contrast, in the section where HS-276 was added following a pre-incubation step with q-FTAA-CN, a large number of labelled Aβ plaques could be seen, but no photons with decay times corresponding to HS-276 were detected. Instead, the emitted photons displayed much shorter decay times (0.30 ns to 0.45 ns) representing q-FTAA-CN emission (Figure 7B). Thus, pre-incubation with q-FTAA-CN prevented HS-276 from binding to Aβ plaques, indicating that q-FTAA-CN and HS-276 share analogous binding sites on these structures.

As HS-169 bound to Aβ deposits^[53]^ has different emission characteristics than HS-276 when bound to Aβ aggregates (Figure S2), the fluorescence decay of HS-276 and HS-169 could be detected by using the same excitation wavelength, 405 nm, as well as two complementary photomultiplier tube (PMT) detectors; one that detected photons at wavelengths below 500 nm (*<*500), and one that detected photons at wavelengths above 500 nm (*>*500). With the *>*500 detector, photons emitted from HS-169 (emission maximum at 670 nm) were detected, and fluorescence decay times between 3.0 ns to 4.1 ns were observed (Figure 7C). Interestingly, photons were also detected with the *<*500 detector implying that HS-276 (emission maximum at 460 nm) was able to bind to Aβ plaques even after pre-incubation with 100-fold higher concentration of HS-169 (Figure 7C). When staining a section with only HS-169 (10 μM), almost no photons could be registered from the Aβ plaques using the *<*500 detector (Figure S5). The decay times of HS-276 were slightly shorter than previously observed (Figure 3B, Figure 7A), indicating that HS-169 might bind in close proximity to HS-276. Overall, the results suggest that HS-276 and HS-169 have different binding modes to Aβ plaques in AD.

To verify the non-competitive or competitive binding mode for HS-276 with the other LCOs, we next compared the binding mode of q-FTAA-CN and HS-169. After pre-incubation with 10 μM HS-169, the section was stained with 100 nM q-FTAA-CN. Similar to the previous experiment, photons emitted from HS-169 were detected in the *>*500 detector and demonstrated comparable fluorescence decay values. With the *<*500 detector, q-FTAA-CN emitted photons could be observed, confirming that q-FTAA-CN, just like HS-276, did not bind to Aβ plaques in the same mode as HS-169 (Figure 7D). Presence of HS-169 seemed to have an effect also on q-FTAA-CN emission as the decay times of the ligand were slightly longer than observed earlier (Figure 7B). Alternatively, the longer decays might be observed as a consequence of the lower concentration, 100 nM instead of 10 μM, being used.

## Discussion

The development of ligands to detect Aβ plaques and tau filamentous inclusions in the brain of AD patients would greatly facilitate the diagnosis of the disease. Having access to molecular tools for monitoring the formation and progression of these pathological hallmarks might also result in important information regarding their role in the pathogenesis of the disease, as well as providing methods for evaluating the effect of therapeutic agents. However, as targets, Aβ plaques and tau lesions are rather different compared to traditional biomolecules such as enzymes and receptors. Several studies have revealed a structural heterogeneity of Aβ deposits,^[24,25,45,59]^ and when high-resolution structures of tau filaments derived from human brains were determined, the results clearly showed a conformational variation of tau in different diseases.^[36–38,40]^ Hence, instead of targeting a defined molecular structure, tracing agents for Aβ and tau aggregates must be directed against targets that display a structural variation. To manage the polymorphic nature of these pathological protein entities and to achieve a complete assessment of aggregate morphotypes, a variety of ligands is needed. Therefore, it is essential to continue the screening for chemical scaffolds that demonstrate a high affinity for aggregates of Aβ or tau. Herein, a small library of ligands was synthesised by combining thiophene-based ligands with the scaffold of the tau tracer MK-6240, which exhibits high selectivity and specificity for NFTs in AD.^[18]^ The binding properties of the resulting structures were evaluated on AD brain tissue as recent studies have shown a morphological difference between synthetic Aβ or tau fibrils and patient-derived aggregates.^[35,39]^ When performing double-staining experiments with the ligand HS-276 and antibodies directed against Aβ or tau, we observed that combining the azaindole motif with a methyl-bithiophene-carboxylate unit instead of the isoquinoline moiety found in MK-6240, resulted in a shift of binding from tau to Aβ. Similarly, in a previous study aimed at identifying potential tracers for Aβ pathology, linking methyl-azaindole with oxazolo[5,4-*b*]pyridine also resulted in high affinity for Aβ plaques in human brain tissue.^[60]^ The methyl-bithiophene-carboxylate moiety found in HS-276 is also present in the structure of b-TVBT2; however, in b-TVBT2, it is not combined with an azaindole motif but with a benzothiazole unit. As b-TVBT2 is highly selective for tau aggregates in AD,^[50]^ the staining results clearly showed that, by replacing benzothiazole with azaindole, a change of the binding site occurred from tau inclusions to Aβ plaques. A similar shift in protein selectivity was also observed when changing the sulphur atom in the benzothiazole unit in the b-TVBT scaffold to a nitrogen.^[50]^ Benzothiazole is also present in the chemical structure of the tau ligand PBB3^[51]^ and when synthesising a structural analogue of PBB3, in which the benzothiazole moiety was substituted with a benzoselenazole constituent, selective binding to Aβ instead of tau was observed.^[61]^ Furthermore, if the linker in PBB3 is shortened and the nitrogen in the pyridine unit is removed, the ligand is transformed into PIB.^[51,62]^ Hence, the preferred binding site can be shifted from tau to Aβ by replacing the benzothiazole unit in the tau selective ligands b-TVBT2 and PBB3, but also by introducing much smaller changes in the chemical structure. Moreover, when connecting benzothiazole with coumarin, nanomolar affinity towards Aβ aggregates was achieved.^[63]^ A coumarin-quinoline conjugate-based near-infrared fluorescence probe, demonstrating high selectivity for Aβ deposits in AD brain tissue sections, has also been reported.^[64]^

Interestingly, HS-276 labelled Aβ cored plaques and diffuse plaques in the grey matter as well as Aβ deposits in the white matter, but the staining of CAA lesions was lacking. In addition, the core of cored plaques displayed much higher intensity of HS-276 emission compared to the diffuse plaques or the halo surrounding the core of cored plaques. An explanation of this might be that the core of the cored plaques consists of dense compact amyloid. Furthermore, cored plaques show intense staining with the amyloid dyes Thioflavin T and Congo red, whereas diffuse plaques are only weakly labelled with these agents.^[65]^ Studies have also shown that parenchymal and vascular Aβ deposits consist of different lengths of the Aβ peptide^[66,67,68]^ suggesting that HS-276 might recognize aggregated Aβ species comprised of specific variants of the Aβ peptide. As recently showed with a multimodal chemical imaging paradigm combining fluorescence microscopy and imaging mass spectroscopy,^[69]^ a tetrameric and a heptameric LCO displayed alternative binding to Aβ deposits comprised of specific variants of Aβ 1–40 or Aβ 1–42. Hence, a similar methodology can most likely be utilized to investigate the diverse binding of HS-276 to different aggregated Aβ pathologies and such experiments are ongoing.

The correlation between aggregate selectivity and minor chemical alterations of the ligands was also noted when rearranging the nitrogen atoms in the azaindole unit of HS-276. Ligands having an azabenzimidazole (HS-302) or a benzimidazole (HS-315) building block instead of the azaindole moity displayed a similar Aβ selectivity as HS-276. In contrast, the indazole containing ligand HS-300 labelled Aβ plaques as well as structures resembling tau aggregates. It was, however, difficult to achieve co-staining with a tau antibody, and a plausible explanation for this might be that the antibody was masking the epitope for HS-300. A similar phenomenon has been reported previously when the labelling of prion aggregates in scrapie-affected sheep brain sections with the thiophene-based ligand PTAA was abrogated by pre-treatment with an antibody.^[47]^ Although the fluorescence intensity from HS-300 was weaker when bound to tau aggregates, the ligand demonstrated co-labelling with b-TVBT2 in AD brain sections, which confirmed that the ligand was binding to tau and that these ligands seemingly have different binding modes to these deposits. Since HS-300 was the only ligand able to bind to tau, the indazole moiety, with a distinct arrangement of nitrogen atoms in the heterocyclic moiety, was considered necessary to afford binding to tau aggregates. Interestingly, when comparing the ligands by thin layer chromatography, HS-300 displayed a more non-polar behaviour than the other ligands, suggesting that a ligand with a certain lipophilicity is necessary to achieve interactions with tau aggregates. The requirements of distinct lipophilicity and amphiphilicity for achieving ligands that are selective towards Aβ or tau aggregates have also been shown for other molecular scaffolds, such as *N*’-benzylidene-benzohydrazide (NBBs) ligands.^[70]^

It has previously been shown that anionic oligothiophenes, or LCOs, bind in a similar mode as Congo red and X-34, and that the binding was highly dependent on interactions between the anionic carboxyl groups of the ligands and the repetitive cationic lysine residues along the fibril surface.^[49,71–73]^ In this study, when introducing an anionic group to the bithiophene moiety of the new thiophene-based ligands, we observed that only HS-301 showed staining of protein deposits, mainly Aβ plaques, in AD brain sections. Thus, in contrast to LCOs, the interaction between the new thiophene-based ligands and the protein aggregate is not dependent on similar electrostatic interactions, suggesting that these ligands have an alternative binding mode than the previously reported oligothiophenes.^[71–73]^ This assumption was also verified when after pre-staining tissue sections with HS-169,^[53]^ an anionic pentameric LCO, labelling of Aβ deposits could still be observed with HS-276, despite using a 100-fold excess of HS-169. In contrast, pre-incubation with the tetrameric oligothiophene q-FTAA-CN^[49]^ completely abolished staining of Aβ aggregates with HS-276, suggesting that these ligands have a similar binding mode to Aβ deposits. A recent study showed that the α-terminal cyano-group found in q-FTAA-CN had a major impact on the ligand’s affinity for Aβ deposits.^[49]^ In fact, when substituting a hydrogen with the cyano-group, the EC_50_ value of the ligand for brain-derived Aβ fibrils decreased from 300–500 nM to less than 0.1 nM.^[49]^ In addition, when introducing the α-terminal cyano-group to the tetrameric thiophene scaffold, an increased selectivity towards Aβ aggregates compared to tau deposits was also afforded in tissue sections with AD pathology.^[49]^ The increased affinity as well as the Aβ aggregate selectivity demonstrated by q-FTAA-CN might indicate that the ligand exhibits an additional binding mode to Aβ deposits compared to other LCOs. Similar to HS-276, q-FTAA-CN also managed to label Aβ aggregates despite pre-incubation with 100-fold excess of the LCO HS-169. Since this binding mode is most likely different than the one exhibited by other ligands such as PIB, X-34 and LCOs, it will be of great interest to employ theoretical calculations to assess the molecular details of the HS-276/q-FTAA-CN specific binding, and such experiments are ongoing. Ligands with alterative binding modes to conventional ligands will be particularly relevant for mapping heterogenic Aβ deposits and examine their role in the pathogenesis of the disease, as well as for developing novel PET tracers that will aid in the clinical diagnosis of AD.

## Conclusion

The discovery of novel methodologies aimed at achieving an early and accurate molecular diagnosis of AD will be crucial to combat the disease. In this regard, as the processes resulting in the formation of the two pathological hallmarks, plaques composed of Aβ aggregates and neurofibrillary tangles made of aggregated tau, presumably occur many years before the clinical symptoms, ligands targeting these formations may have great potential as diagnostic tools. Abundant evidence argues that Aβ and tau form aggregates with distinct conformations, which give rise to different pathological properties and clinical features. Thus, to be able to detect the large variation of aggregate morphotypes, a variety of ligands will be needed. Herein, we have developed a new thiophene-based molecular scaffold that selectively target Aβ aggregates in AD. The selectivity was highly dependent on the molecular composition of the ligand, and experiments indicated alternate binding mode compared to ligands introduced earlier. Our findings provide useful knowledge of how small changes of the structure of the ligand influence its binding properties. This knowledge may potentially aid in the design of new ligands that can distinguish between different pathogenic protein aggregates and expand the toolbox of ligands that can be utilized to assign distinct aggregated species of Aβ. The latter would be highly relevant both from a clinical perspective, as well as for explaining the pathological relevance of specific Aβ aggregates in AD.

### Experimental Section

Full experimental details including additional characterisation data and NMR spectra of new ligands, as well as supporting figures, are given in the Supporting Information. Frozen brain tissue from a neuropathologically confirmed case of AD was obtained from the Dementia Laboratory at the Department of Pathology and Laboratory Medicine, Indiana University School of Medicine, Indianapolis, USA. The studies carried out at the Indiana University School of Medicine were reviewed and approved by the Indiana University Institutional Review Board and informed consent was obtained from the patient or their next of kin. The experiments performed at Linköping University were reviewed and approved by a national ethical committee (approval number 2020–01197).

## Supplementary Material

Supplementary material

## References

[1] Winblad B, Amouyel P, Andrieu S, Ballard C, Brayne C, Brodaty H, Cedazo-Minguez A, Dubois B, Edvardsson D, Feldman H, Fratiglioni L, Frisoni GB, Gauthier S, Georges J, Graff C, Iqbal K, Jessen F, Johansson G, Jönsson L, Kivipelto M, Knapp M, Mangialasche F, Melis R, Nordberg A, Olde Rikkert M, Qiu C, Sakmar TP, Scheltens P, Schneider LS, Sperling R, Tjernberg LO, Waldemar G, Wimo A, Zetterberg H, Lancet Neurol. 2016, 15, 455–532. 26987701 10.1016/S1474-4422(16)00062-4

[2] Bateman RJ, Xiong C, Benzinger TLS, Fagan AM, Goate A, Fox NC, Marcus DS, Cairns NJ, Xie X, Blazey TM, Holtzman DM, Santacruz A, Buckles V, Oliver A, Moulder K, Aisen PS, Ghetti B, Klunk WE, McDade E, Martins RN, Masters CL, Mayeux R, Ringman JM, Rossor MN, Schofield PR, Sperling RA, Salloway S, Morris JC, Engl N. J. Med. 2012, 367, 795–804. 10.1056/NEJMoa1202753 PMC3474597 22784036

[3] Braak H, Braak E, Neurobiol. Aging. 1997, 18, 351–357. 9330961 10.1016/s0197-4580(97)00056-0

[4] Jack CR, Lowe VJ, Weigand SD, Wiste HJ, Senjem ML, Knopman DS, Shiung MM, Gunter JL, Boeve BF, Kemp BJ, Weiner M, Petersen RC, Brain 2009, 132, 1355–1365.19339253

[5] Jack CR, Knopman DS, Jagust WJ, Shaw LM, Aisen PS, Weiner MW, Petersen RC, Trojanowski JQ, Lancet Neurol. 2010, 9, 119–128. 20083042 10.1016/S1474-4422(09)70299-6 PMC2819840

[6] Price JL, Morris JC, Ann. Neurol. 1999, 45, 358–368. 10072051 10.1002/1531-8249(199903)45:3<358::aid-ana12>3.0.co;2-x

[7] Klunk WE, Engler H, Nordberg A, Wang Y, Blomqvist G, Holt DP, Bergström M, Savitcheva I, Huang G-F, Estrada S, Ausén B, Debnath ML, Barletta J, Price JC, Sandell J, Lopresti BJ, Wall A, Koivisto P, Antoni G, Mathis CA, Långström B, Ann. Neurol. 2004, 55, 306–319. 14991808 10.1002/ana.20009

[8] Cselényi Z, Erikdotter Jönhagen M, Forsberg A, Halldin C, Julin P, Schou M, Johnström P, Varnäs K, Svensson S, Farde L, J. Nucl. Med. 2012, 53, 415–424. 22323782 10.2967/jnumed.111.094029

[9] Rowe CC, Ackerman U, Browne W, Mulligan R, Pike KL, O’Keefe G, Tochon-Danguy H, Chan G, Berlangieri SU, Jones G, Dickinson-Rowe KL, Kung HP, Zhang W, Kung M-P, Skovronsky D, Dyrks T, Holl G, Krause S, Friebe M, Lehman L, Lindemann S, Dinkelborg LM, Masters CL, Villemagne VL, Lancet Neurol. 2008, 7, 129–135. 18191617 10.1016/S1474-4422(08)70001-2

[10] Vanderberghe R, Van Laere K, Ivanoiu A, Salmon E, Bastin C, Triau E, Hasselbalch S, Law I, Andersen A, Korner A, Minthon L, Garraux G, Nelissen N, Bormans G, Buckley C, Owenius R, Thurfjell L, Farrar G, Brooks DJ, Ann. Neurol. 2010, 68, 319–329. 20687209 10.1002/ana.22068

[11] Wong DF, Rosenberg PB, Zhou Y, Kumar A, Raymont V, Ravert HT, Dannals RF, Nandi A, Brašic JR, Ye W, Hilton J, Lyketsos C, Kung HF, Joshi AD, Skovronsky DM, Pontecorvo MJ, J. Nucl. Med. 2010, 51, 913–920. 20501908 10.2967/jnumed.109.069088 PMC3101877

[12] Lemoine L, Gillberg PG, Svedberg M, Stepanov V, Jia Z, Huang J, Nag S, Tian H, Ghetti B, Okamura N, Higuchi M, Halldin C, Nordberg A, Alzheimer’s Res. Ther. 2017, 9, 96. 29229003 10.1186/s13195-017-0325-z PMC5725799

[13] Leuzy A, Chiotis K, Lemoine L, Gillberg P-G, Almkvist O, Rodriguez-Vieitez E, Nordberg A, Mol. Psychiatry 2019, 24, 1112–1134. 30635637 10.1038/s41380-018-0342-8 PMC6756230

[14] Ng KP, Pascoal TA, Mathotaarachchi S, Therriault J, Kang MS, Shin M, Guiot M-C, Guo Q, Harada R, Comley RA, Massarweh G, Soucy J-P, Okamura N, Gauthier S, Rosa-Neto P, Alzheimer’s Res. Ther. 2017, 9, 25. 28359327 10.1186/s13195-017-0253-y PMC5374697

[15] Vermeiren C, Motte P, Viot D, Mairet-Coello G, Courade J-P, Citron M, Mercier J, Hannestad J, Gillard M, Mov. Disord. 2018, 33, 272–281. 10.1002/mds.27271 29278274

[16] Villemagne VL, Fodero-Tavoletti M, Masters CL, Rowe CC, Lancet Neurol. 2015, 14, 114–124. 25496902 10.1016/S1474-4422(14)70252-2

[17] Kroth H, Oden F, Molette J, Schieferstein H, Capotosti F, Mueller A, Berndt M, Schmitt-Willich H, Darmency V, Gabellieri E, Boudou C, Juergens T, Varisco Y, Vokali E, Hickman DT, Tamagnan G, Pfeifer A, Dinkelborg L, Muhs A, Stephens A, Eur. J. Nucl. Med. Mol. Imaging. 2019, 46, 2178–2189. 31264169 10.1007/s00259-019-04397-2 PMC6667408

[18] Walji AM, Hostetler ED, Selnick H, Zeng Z, Miller P, Bennacef I, Salinas C, Connolly B, Gantert L, Holahan M, O’Malley S, Purcell M, Riffel K, Li J, Balsells J, OBrien JA, Melquist S, Soriano A, Zhang X, Ogawa A, Xu S, Joshi E, Della Rocca J, Hess FJ, Schachter J, Hesk D, Schenk D, Struyk A, Babaoglu K, Lohith TG, Wang Y, Yang K, Fu J, Evelhoch JL, Coleman PJ, J. Med. Chem. 2016, 59, 4778–4789. 27088900 10.1021/acs.jmedchem.6b00166

[19] Aguero C, Dhaynaut M, Normandin MD, Amaral AC, Guehl NJ, Neelamegam R, Marquie M, Johnson KA, El Fakhri G, Frosch MP, Gómez-Isla T, Acta Neuropathol. 2019, 7, 37. 10.1186/s40478-019-0686-6 PMC6410510 30857558

[20] Marquié M, Normandin MD, Meltzer AC, Siao Tick Chong M, Andrea NV, Antón-Fernández A, Klunk WE, Mathis CA, Ikonomovic MD, Debnath M, Bien EA, Vanderburg CR, Constantino I, Makaretz S, DeVos SL, Oakley DH, Gomperts SN, Growdon JH, Domoto-Reilly K, Lucente D, Dickerson BC, Frosch MP, Hyman BT, Johnson KA, Gómez-Isla T, Ann. Neurol. 2017, 81, 117–128. 27997036 10.1002/ana.24844 PMC5319193

[21] Rosen RF, Ciliax BJ, Wingo TS, Gearing M, Dooyema J, Lah JJ, Ghiso JA, LeVine H, Walker LC, Acta Neuropathol. 2010, 119, 221–233. 19690877 10.1007/s00401-009-0583-3 PMC3045810

[22] Schöll M, Wall A, Thordardottir S, Ferreira D, Bogdanovic N, Långström B, Almkvist O, Graff C, Nordberg A, Neurology 2012, 79, 229–236. 22700814 10.1212/WNL.0b013e31825fdf18

[23] Collinge J, Clarke AR, Science. 2007, 318, 930–936. 17991853 10.1126/science.1138718

[24] Condello C, Lemmin T, Stöhr J, Nick M, Wu Y, Maxwell AM, Watts JC, Caro CD, Oehler A, Keene CD, Bird TD, van Duinen SG, Lannfelt L, Ingelsson M, Graff C, Giles K, DeGrado WF, Prusiner SB, Proc. Natl. Acad. Sci. USA 2018, 115, E782–E791. 29311311 10.1073/pnas.1714966115 PMC5789926

[25] Lu J-X, Qiang W, Yau W-M, Schwieters CD, Meredith SC, Tycko R, Cell 2013,154, 1257–1268. 24034249 10.1016/j.cell.2013.08.035 PMC3814033

[26] Meyer-Luehmann M, Coomaraswamy J, Bolmont T, Kaeser S, Schaefer C, Kilger E, Neuenschwander A, Abramowski D, Frey P, Jaton AL, Vigouret JM, Paganetti P, Walsh DM, Mathews PM, Ghiso J, Staufenbiel M, Walker LC, Jucker M, Science 2006, 313, 1781–1784. 16990547 10.1126/science.1131864

[27] Maarouf CL, Daugs ID, Spina S, Vidal R, Kokjohn TA, Patton RL, Kalback WM, Luehrs DC, Walker DG, Castaño EM, Beach TG, Ghetti B, Roher AE, Mol. Neurodegener. 2008, 3, 20. 19021905 10.1186/1750-1326-3-20 PMC2600784

[28] Petkova AT, Leapman RD, Guo Z, Yau W-M, Mattson MP, Tycko R, Science 2005, 307, 262–265. 15653506 10.1126/science.1105850

[29] Stöhr J, Condello C, Watts JC, Bloch L, Oehler A, Nick M, DeArmond SJ, Giles K, DeGrado WF, Prusiner SB, Proc. Natl. Acad. Sci. USA 2014, 111, 10329–10334. 24982137 10.1073/pnas.1408968111 PMC4104853

[30] Watts JC, Condello C, Stöhr J, Oehler A, Lee J, DeArmond SJ, Lannfelt L, Ingelsson M, Giles K, Prusiner SB, Proc. Natl. Acad. Sci. USA 2014, 111, 10323–10328. 24982139 10.1073/pnas.1408900111 PMC4104857

[31] Crowther RA, Goedert M, J. Struct. Biol. 2000, 130, 271–279. 10940231 10.1006/jsbi.2000.4270

[32] Guo JL, Narasimhan S, Changolkar L, He Z, Stieber A, Zhang B, Gathagan RJ, Iba M, McBride JD, Trojanowski JQ, Lee VMY, J. Exp. Med. 2016, 213, 2635–2654. 27810929 10.1084/jem.20160833 PMC5110027

[33] Taniguchi-Watanabe S, Arai T, Kametani F, Nonaka T, Masuda-Suzukake M, Tarutani A, Murayama S, Saito Y, Arima K, Yoshida M, Akiyama H, Robinson A, Mann DMA, Iwatsubo T, Hasegawa M, Acta Neuropathol. 2016, 131, 267–280. 26538150 10.1007/s00401-015-1503-3 PMC4713716

[34] Xu H, O’Reilly M, Gibbons GS, Changolkar L, McBride JD, Riddle DM, Zhang B, Stieber A, Nirschl J, Kim S-J, Hoxha K-H, Brunden KR, Schellenberg GD, Trojanowski JQ, Lee VMY, Acta Neuropathol. 2020, 141, 193–215. 10.1007/s00401-020-02253-4 PMC7847465 33385254

[35] Kollmer M, Close W, Funk L, Rasmussen J, Bsoul A, Schierhorn A, Schmidt M, Sigurdson CJ, Jucker M, Fändrich M, Nat. Commun. 2019, 10, 4760. 31664019 10.1038/s41467-019-12683-8 PMC6820800

[36] Falcon B, Zhang W, Murzin AG, Murshudov G, Garringer HJ, Vidal R, Crowther RA, Ghetti B, Scheres SHW, Goedert M, Nature. 2019, 561, 137–140. 10.1038/s41586-018-0454-y PMC6204212 30158706

[37] Falcon B, Zivanov J, Zhang W, Murzin AG, Garringer HJ, Vidal R, Crowther RA, Newell KL, Ghetti B, Goedert M, Scheres SHW, Nature 2019, 568, 420–423. 30894745 10.1038/s41586-019-1026-5 PMC6472968

[38] Fitzpatrick AWP, Falcon B, He S, Murzin AG, Murshudov G, Garringer HJ, Crowther RA, Ghetti B, Goedert M, Scheres SHW, Nature 2017, 547, 185–190. 28678775 10.1038/nature23002 PMC5552202

[39] Zhang W, Falcon B, Murzin AG, Fan J, Crowther RA, Goedert M, Scheres SHW, eLife 2019, 8, e43584. 10.7554/eLife.43584 PMC6375701 30720432

[40] Zhang W, Tarutani A, Newell KL, Murzin AG, Matsubara T, Falcon B, Vidal R, Garringer HJ, Shi Y, Ikeuchi T, Murayama S, Ghetti B, Hasegawa M, Goedert M, Scheres SHW, Nature 2020, 580, 283–287. 32050258 10.1038/s41586-020-2043-0 PMC7148158

[41] Klingstedt T, Åslund A, Simon RA, Johansson LBG, Mason JJ, Nyström S, Hammarström P, Nilsson KPR, Org. Biomol. Chem. 2011, 9, 8356–8370. 22051883 10.1039/c1ob05637a PMC3326384

[42] Klingstedt T, Shirani H, Mahler J, Wegenast-Braun BM, Nyström S, Goedert M, Jucker M, Nilsson KPR, Chem. Eur. J. 2015, 21, 9072–9082. 26013403 10.1002/chem.201500556 PMC4517144

[43] Klingstedt T, Ghetti B, Holton JL, Ling H, Nilsson KPR, Goedert M, Acta Neuropathol. 2019, 7, 193. 10.1186/s40478-019-0840-1 PMC6892142 31796099

[44] Nyström S, Psonka-Antonczyk KM, Ellingsen PG, Johansson LBG, Reitan N, Handrick S, Prokop S, Heppner FL, Wegenast-Braun BM, Jucker M, Lindgren M, Stokke BT, Hammarström P, Nilsson KPR, ACS Chem. Biol. 2013, 8, 1128–1133. 23521783 10.1021/cb4000376

[45] Rasmussen J, Mahler J, Beschorner N, Kaeser SA, Häsler LM, Baumann F, Nyström S, Portelius E, Blennow K, Lashley T, Fox NC, Sepulveda-Falla D, Glatzel M, Oblak AL, Ghetti B, Nilsson KPR, Hammarström P, Staufenbiel M, Walker LC, Jucker M, Proc. Natl. Acad. Sci. USA 2017, 114, 13018–130123. 29158413 10.1073/pnas.1713215114 PMC5724274

[46] Shahnawaz M, Mukherjee A, Pritzkow S, Mendez N, Rabadia P, Liu X, Hu B, Schmeichel A, Singer W, Wu G, Tsai A-L, Shirani H, Nilsson KPR, Low PA, Soto C, Nature 2020, 578, 273–277. 32025029 10.1038/s41586-020-1984-7 PMC7066875

[47] Sigurdson CJ, Nilsson KPR, Hornemann S, Manco G, Polymenidou M, Schwarz P, Leclerc M, Hammarström P, Wüthrich K, Aguzzi A, Nat. Methods 2007, 4, 1023–1030. 18026110 10.1038/nmeth1131

[48] Åslund A, Sigurdson CJ, Klingstedt T, Grathwohl S, Bolmont T, Dickstein DL, Glimsdal E, Prokop S, Lindgren M, Konradsson P, Holtzman DM, Hof PR, Heppner FL, Gandy S, Jucker M, Aguzzi A, Hammarström P, Nilsson KPR, ACS Chem. Biol. 2009, 4, 673–684. 19624097 10.1021/cb900112v PMC2886514

[49] Bäck M, Appelqvist H, LeVine H, Nilsson KPR, Chem. Eur. J. 2016, 22, 18335–18338. 27767229 10.1002/chem.201604583 PMC5215536

[50] Shirani H, Appelqvist H, Bäck M, Klingstedt T, Cairns NJ, Nilsson KPR, Chem. Eur. J. 2017, 23, 17127–17135. 28926133 10.1002/chem.201703846 PMC5928317

[51] Maruyama M, Shimada H, Suhara T, Shinotoh H, Ji B, Maeda J, Zhang M-R, Trojanowski JQ, Lee VMY, Ono M, Masamoto K, Takano H, Sahara N, Iwata N, Okamura N, Furumoto S, Kudo Y, Chang Q, Saido TC, Takashima A, Lewis J, Jang M-K, Aoki I, Ito H, Higuchi M, Neuron 2013, 79, 1094–1108. 24050400 10.1016/j.neuron.2013.07.037 PMC3809845

[52] Antilla JC, Baskin JM, Barder TE, Buchwald SL, J. Org. Chem. 2004, 69, 5578–5587. 15307726 10.1021/jo049658b

[53] Shirani H, Linares M, Sigurdson CJ, Lindgren M, Norman P, Nilsson KPR, Chem. Eur. J. 2015, 21, 15133–15137. 26388448 10.1002/chem.201502999 PMC4641461

[54] Terman A, Brunk UT, Int. J. Biochem. Cell Biol. 2004, 36, 1400–1404. 15147719 10.1016/j.biocel.2003.08.009

[55] Brunk UT, Terman A, Free Radical Biol. Med. 2002, 33, 611–619. 12208347 10.1016/s0891-5849(02)00959-0

[56] Dowson JH, Histopathology 1981, 5, 305–310. 7016712 10.1111/j.1365-2559.1981.tb01789.x

[57] Thal DR, Ghebremedhin E, Haass C, Schultz C, Clin. Neuropathol. 2002, 21, 35–40. 11846043

[58] Calvo-Rodriguez M, Hou SS, Snyder AC, Dujardin S, Shirani H, Nilsson KPR, Bacskai BJ, Acta Neuropathol. 2019, 7, 171. 10.1186/s40478-019-0832-1 PMC6839235 31703739

[59] Cohen ML, Kim C, Haldiman T, ElHag M, Mehndiratta P, Pichet T, Lissemore F, Shea M, Cohen Y, Chen W, Blevins J, Appleby BS, Surewicz K, Surewicz WK, Sajatovic M, Tatsuoka C, Zhang S, Mayo P, Butkiewicz M, Haines JL, Lerner AJ, Safar JG, Brain. 2015, 138, 1009–1022. 25688081 10.1093/brain/awv006 PMC5014074

[60] Harrison ST, Mulhearn J, Wolkenberg SE, Miller PJ, O’Malley SS, Zeng Z, Williams DL, Hostetler ED, Sanabria-Bohórquez S, Gammage L, Fan H, Sur C, Culberson JC, Hargreaves RJ, Cook JJ, Hartman GD, Barrow JC, ACS Med. Chem. Lett. 2011, 2, 498–502. 24900338 10.1021/ml200018n PMC4018077

[61] Sundaram GSM, Dhavale DD, Prior JL, Yan P, Cirrito J, Rath NP, Laforest R, Cairns NJ, Lee J-M, Kotzbauer PT, Sharma V, Sci. Rep. 2016, 6, 35636. 27805057 10.1038/srep35636 PMC5090206

[62] Mathis CA, Wang Y, Holt DP, Huang G-F, Debnath ML, Klunk WE, J. Med. Chem. 2003, 46, 2740–2754. 12801237 10.1021/jm030026b

[63] Rajasekhar K, Narayanaswamy N, Arul Murugan N, Kuang G, Ågren H, Govindaraju T, Sci. Rep. 2016, 6, 23668. 27032526 10.1038/srep23668 PMC4817056

[64] Rajasekhar K, Narayanaswamy N, Arul Murugan N, Viccaro K, Lee H-G, Shah K, Govindaraju T, Biosens. Bioelectron. 2017, 98, 54–61. 28649025 10.1016/j.bios.2017.06.030 PMC6370041

[65] DeTure MA, Dickson DW, Mol. Neurodegener. 2019, 14, 32. 31375134 10.1186/s13024-019-0333-5 PMC6679484

[66] Gkanatsiou E, Portelius E, Toomey CE, Blennow K, Zetterberg H, Lashley T, Brinkmalm G, Neurosci. Lett. 2019, 701, 125–131. 30807796 10.1016/j.neulet.2019.02.033

[67] Mann DMA, Iwatsubo T, Ihara Y, Cairns NJ, Lantos PL, Bogdanovic N, Lannfelt L, Winblad B, Maat-Schieman MLC, Rossor MN, Am. J. Pathol. 1996, 148, 1257–1266. 8644866 PMC1861527

[68] Prelli F, Castaño E, Glenner GG, Frangione B, Neurochem J. 1988, 51, 648–651. 10.1111/j.1471-4159.1988.tb01087.x 3292706

[69] Michno W, Nyström S, Wehrli P, Lashley T, Brinkmalm G, Guerard L, Syvänen S, Sehlin D, Kaya I, Brinet D, Nilsson KPR, Hammarström P, Blennow K, Zetterberg H, Hanrieder JJ, J. Biol. Chem. 2019, 294, 6719–6732. 30814252 10.1074/jbc.RA118.006604 PMC6497931

[70] Taghavi A, Nasir S, Pickhardt M, Heyny von Haussen R, Mall G, Mandelkow E, Mandelkow E-M, Schmidt B, J. Alzheimer’s Dis. 2011, 27, 835–843. 21891864 10.3233/JAD-2011-111238

[71] Herrmann US, Schütz AK, Shirani H, Huang D, Saban D, Nuvolone M, Li B, Ballmer B, Åslund AKO, Mason JJ, Rushing E, Budka H, Nyström S, Hammarström P, Böckmann A, Caflisch A, Meier BH, Nilsson KPR, Hornemann S, Aguzzi A, Sci. Transl. Med. 2015, 7, 1923. 10.1126/scitranslmed.aab1923 26246168

[72] König C, Skånberg R, Hotz I, Ynnerman A, Norman P, Linares M, Chem. Commun. 2018, 54, 3030–3033. 10.1039/c8cc00105g 29512664

[73] Schütz AK, Hornemann S, Wälti MA, Greuter L, Tiberi C, Cadalbert R, Gantner M, Riek R, Hammarström P, Nilsson KPR, Böckmann A, Aguzzi A, Meier BH, ACS Chem. Neurosci. 2018, 9, 475–481. 29178774 10.1021/acschemneuro.7b00397

