# Supplementary material for: Thiophene-Based Optical Ligands That Selectively Detect Aβ Pathology in Alzheimer’s Disease
Source: Chembiochem. Author manuscript; Available in PMC 2021 Sep 1. (PMC8409278; doi:10.1002/cbic.202100199)
Supplement: Supplementary material [file NIHMS1729252-supplement-Supplementary_material.pdf]

ChemBioChem

Supporting Information

Thiophene-Based Optical Ligands That Selectively Detect Aβ Pathology in Alzheimer's Disease

Therése Klingstedt, Hamid Shirani, Bernardino Ghetti, Ruben Vidal, and K. Peter R. Nilsson*

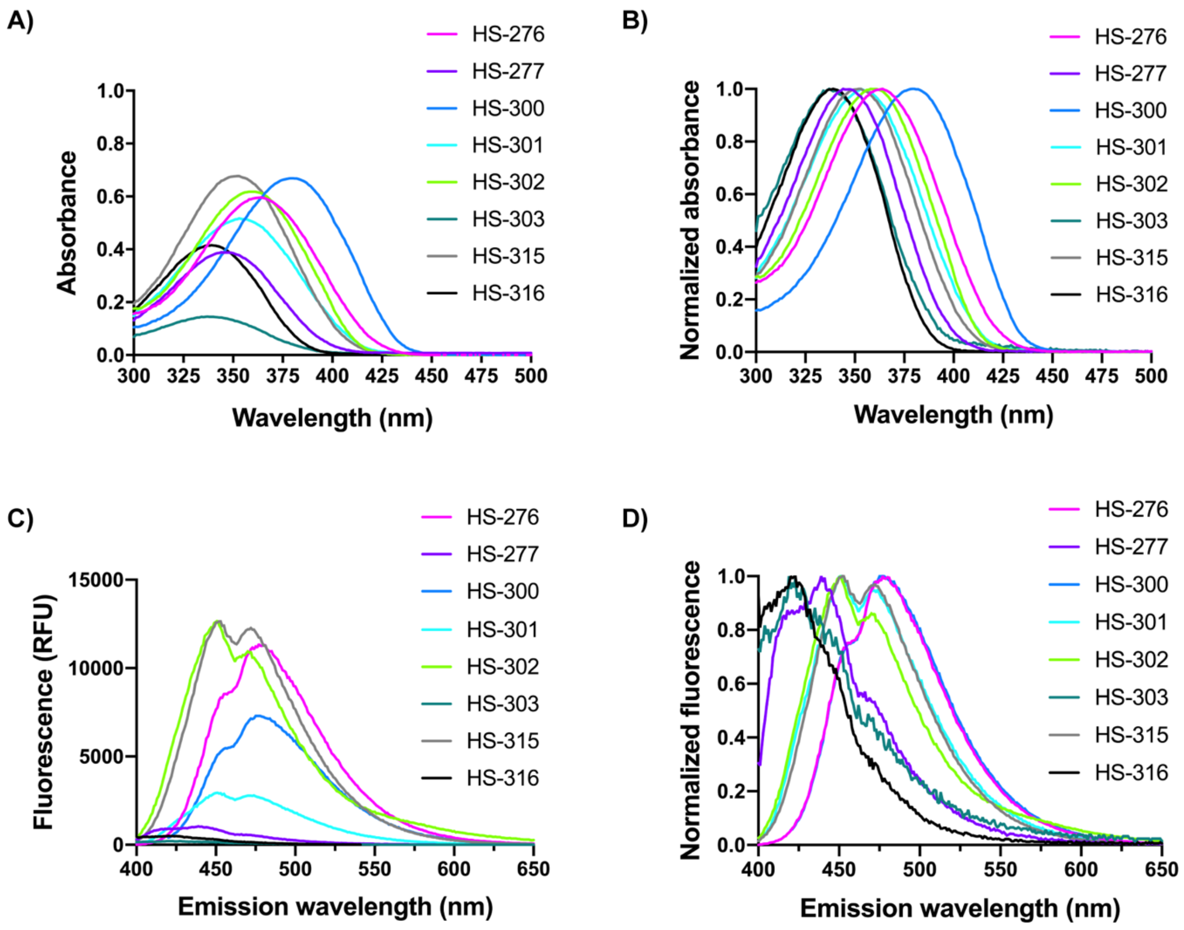

Figure S1. Optical characterisation of thiophene-based ligands mimicking MK-6240. **A)** Absorbance spectrum of ligands dissolved and diluted to 50 μM in DMSO (HS-276, HS-277, HS-300, HS-302, HS-315, HS-316), 50% DMSO (HS-303) or water (HS-301). **B)** Normalization of graph in (A). **C)** Emission spectrum of ligands dissolved and diluted to 600 nM in DMSO (HS-276, HS-277, HS-300, HS-302, HS-315, HS-316), 50% DMSO (HS-303) or water (HS-301). **D)** Normalization of graph in (C).

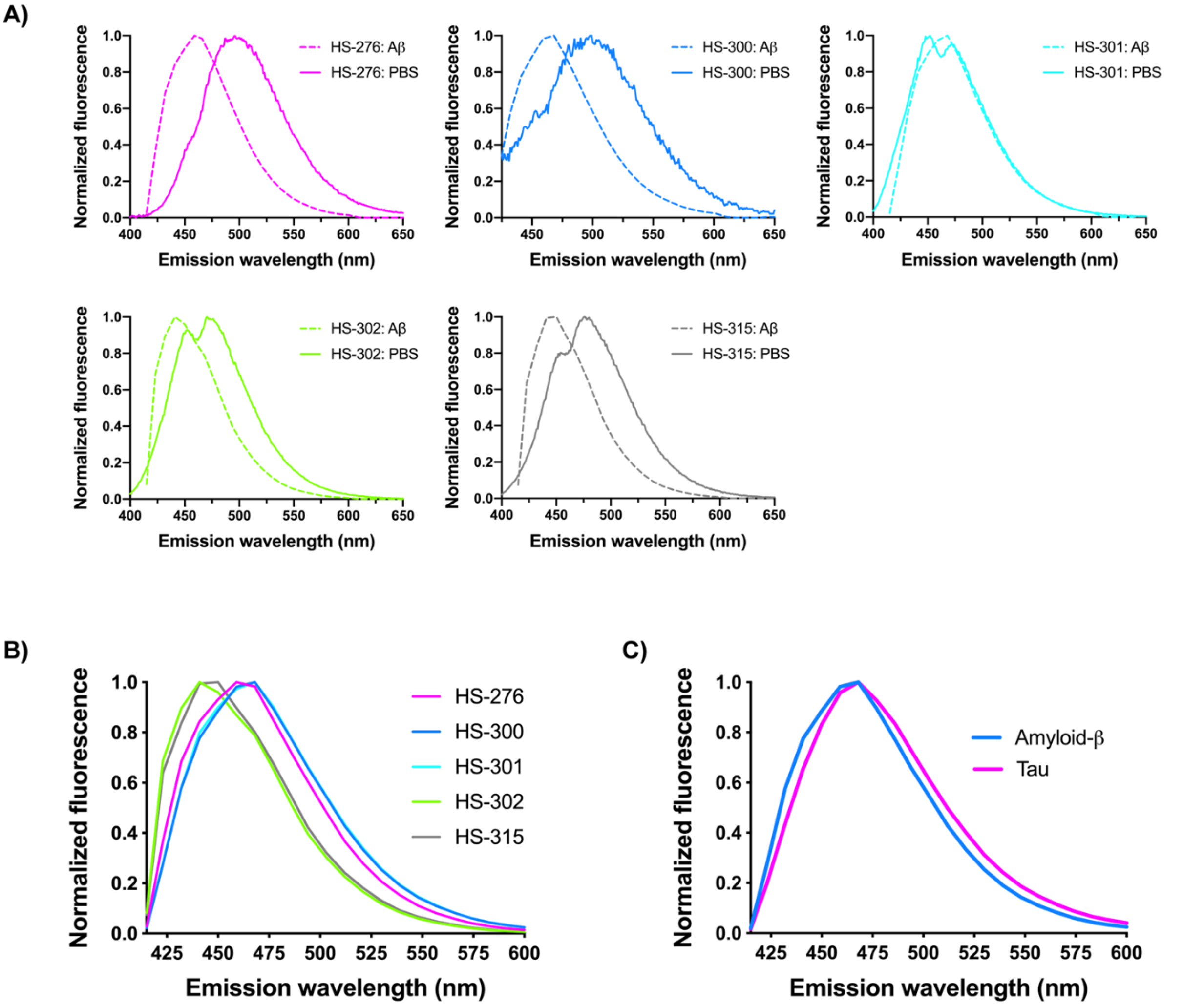

Figure S2. Spectral characterisation of ligands in solution or binding to Aβ plaques or tau aggregates in AD. **A)** Emission spectrum of the indicated ligand in PBS buffer (solid line) or when binding to Aβ plaques in AD brain tissue section (dashed line). **B)** Fluorescence emission spectrum of each ligand when binding to Aβ plaques in AD brain tissue section. All ligands were excited at 405 nm. **C)** Fluorescence emission spectrum of ligand HS-300 when binding to Aβ deposits or tau pathologies in AD brain tissue section. The ligand was excited at 405 nm.

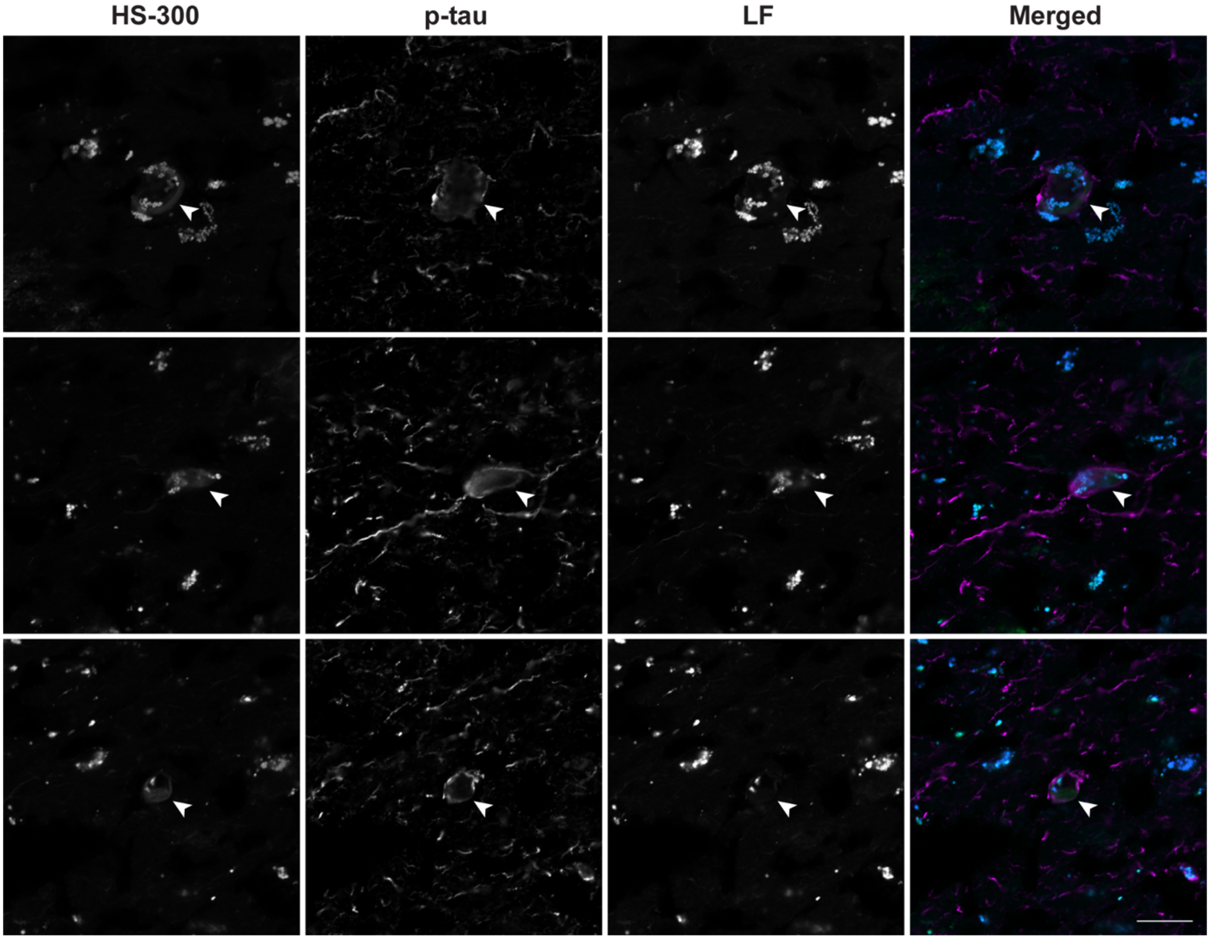

Figure S3. HS-300 shows co-localisation with anti-phospho-tau antibody. Fluorescence images of AD brain tissue section double-stained with HS-300 and anti-phospho-tau antibody (p-tau, AT8). HS-300 showed co-localisation with the antibody confirming that this ligand, in addition to Aβ plaques, also labels tau inclusions. Channels for single staining are depicted in white to enhance visualization. Autofluorescence from lipofuscin (LF) is also shown. Scalebar, 20 μm.

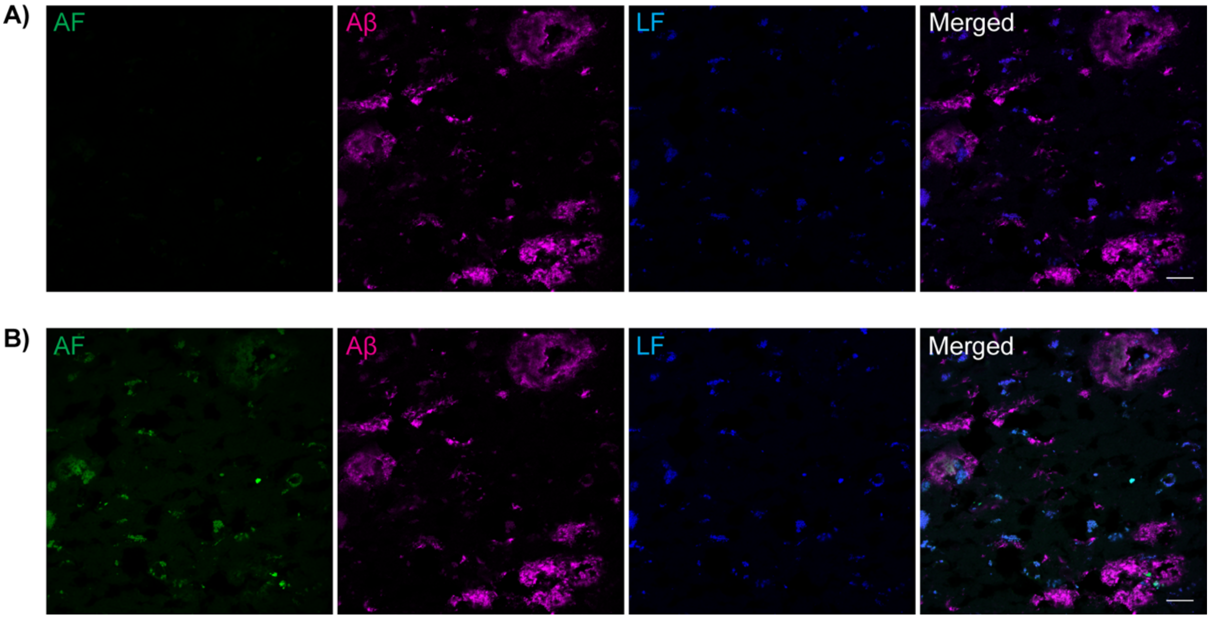

Figure S4. Autofluorescence from Aβ plaques***.*** **A,B)** Fluorescence images of AD brain tissue section stained with anti-Aβ-antibody (magenta, 6E10) showing autofluorescence (AF) from immuno-positive Aβ plaques in green and lipofuscin (LF) in blue. The image was acquired applying the same settings that were used when collecting images of ligand and anti-Aβ-antibody double-stained sections (see Figure 6). Scalebar, 20 μm. **A)** Autofluorescence from Aβ plaques when exciting the sample using the lowest excitation intensity that was used in Figure 6. **B)** Autofluorescence from plaques when exciting the sample using the highest excitation intensity that was used in Figure 6.

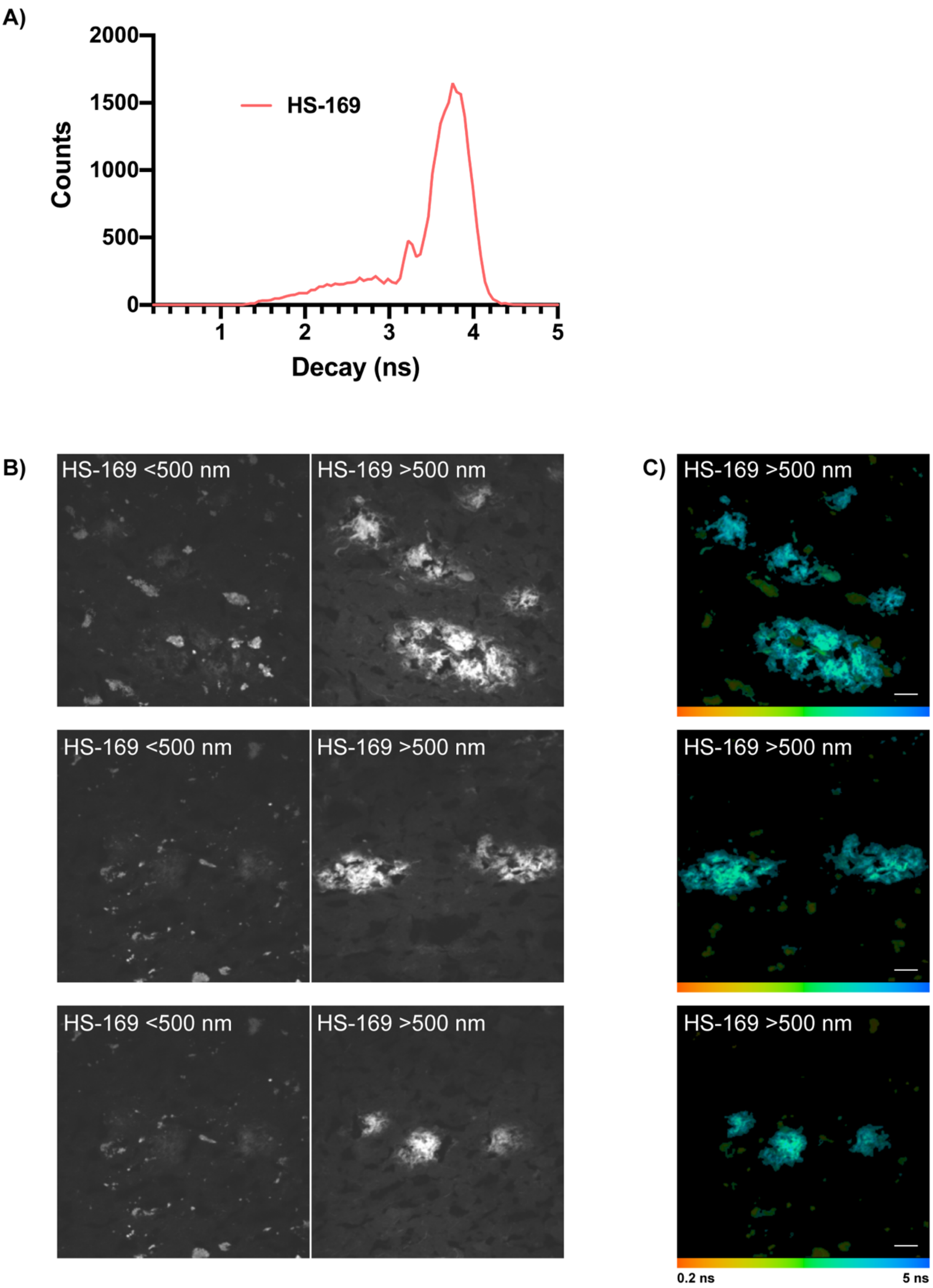

Figure S5. Fluorescence lifetime imaging of HS-169 labelled AD brain section. **A)** Intensity-weighted mean lifetime distribution (t_i) of HS-169 binding to Aβ deposits in AD brain tissue. **B)** Fluorescence intensity images of AD brain tissue section stained with 10 μM HS-169. PMT detectors registering photons with wavelengths below 500 nm (<500) or above 500 nm (>500) were used. With the former, almost no photons were detected, whereas with the latter, a strong fluorescence intensity could be seen from the Aβ plaques. **C)** Fluorescence lifetime images of the region shown in B. The colour-bar

represents lifetimes from 0.2 ps to 5 ps and the images are colour-coded according to lifetime. Scalebar, 20 μm.

Experimental Details

Ligand synthesis

General information

All reagents and solvents were purchased from commercial sources and used as received without further purification. NMR spectra were recorded on a Varian 500 instrument (Varian Inc., Santa Clara, CA, USA) operating at 500 MHz for ^{1}H and 126 MHz for ^{13}C, using the residual solvent signal as reference. The IR spectra were performed on an Avatar 330 FT-IR spectrometer (Thermo Nicolet). TLC was carried out on Merck pre-coated 60 F254 aluminum plates using UV-light (λ = 254 nm and 366 nm) for visualization and column chromatography was carried out on silica gel Merck 60 (40–63 μm). Analytical liquid chromatography/mass spectrometry (LC/MS) was performed on a Waters system equipped with a Waters 1525 gradient pump, 2998 Photodiode Array Detector, 2424 Evaporative Light Scattering Detector, SQD 2 Mass Detector and an Xbridge® C18 column (4.6 x 50 mm, 3.5 μm). Microwave reaction was performed using Biotage® Initiator microwave system. Purification was accomplished by Biotage® Selekt automated flash system through Biotage® Sfär silica columns.

General procedure for N-arylation reaction of 4a-4d (G1)

A mixture of **1a-1d** (1 equiv.), 2,5-dibromothiophene (**2**) (1 equiv.), Cs_2CO_3 (2 equiv.) and CuI (0.2 equiv.) in DMF (2 mL) were added to oven dried reaction glass vial, back filled with nitrogen, sealed with cap and the reaction mixture was heated at 180 °C in microwave cavity for 30 min. After cooling to room temperature, water was added (30 mL) and the mixture extracted with ethyl acetate (2×30 mL). The combined organic layer was washed with water (2×30 mL) and brine (30 mL), dried over $MgSO_4$ and the solvent was removed under reduced pressure. The crude product was subjected to column chromatography yielding **4a-4d**.

General procedure for N-arylation reaction of HS-276, HS-300, HS-302 and HS-315 (G2)

A mixture of **1a-1d** (2 equiv.), dimer **3** (1 equiv.), K_3PO_4 (2 equiv.), N,N′-Dimethylethylenediamine

(0.2 equiv.) and CuI (0.3 equiv.) in toluene (3 mL) were added to oven dried reaction vial, back filled with nitrogen and the reaction tube was sealed with cap and heated at 110 °C in oil bath for 24h. After cooling to room temperature, water was added (30 mL) and the mixture extracted with ethyl acetate (2×30 mL). The combined organic layer was washed with water (2×30 mL) and brine (30 mL), dried over $MgSO_4$ and the solvent was removed under reduced pressure. The crude product was subjected to column chromatography yielding **HS-276, HS-300, HS-302 and HS-315**.

General procedure for Suzuki coupling (G3)

A mixture of the **4a-4d**, methyl 5-(4,4,5,5-tetramethyl-1,3,2-dioxaborolan-2-yl) thiophene-2-carboxylate (**5**) (1.2 equiv.), K_2CO_3 (2 equiv./bromine, in 1,4-dioxane/methanol (8:2, 8 mL/mmol, degassed) and PEPPS^{TM}-IPr (5 mol %) was heated to 80 °C for 1-3 h. After cooling to room temperature pH was adjusted to 4 by HCl (1M) and the residue was extracted with chloroform (3×30 mL/mmol), washed with water (2×30 mL/mmol) and brine (30 mL). The combined organic phase was dried over $MgSO_4$ and the solvent was evaporated. The crude product was purified by column chromatography to give desired products **HS-276, HS-300, HS-302 and HS-315**.

General procedure for hydrolysis (G4)

LiOH (3 M, 4 equiv./ester) was added to a solution of the **HS-276, HS-300, HS-302 and HS-315** in 1,4-dioxane/H_2O (9:1) and heated to 80 °C until LC-MS indicated completion of hydrolysis. HCl (1 M, aq.) was added, and the precipitate was collected by centrifugation. The precipitant was washed with water, dried and subsequently converted to its corresponding sodium salt by dissolving it in 1,4-dioxane/H_2O (2:1) and NaOH (1 M, 1.5 equiv./ester). The solution was freeze-dried to afford products **HS-277, HS-301, HS-303 and HS-316.**

4a

General procedure of N-arylation reactions **G1** was applied starting with **1a** (49 mg, 0.41 mmol) and **2** (100 mg, 0.41 mmol). The residue was subjected to column chromatography using [heptane/acetone

(0→20 %)] to give **4a** (40 mg, 35 %) as white solid. IR (neat) 1549, 1499, 1464, 1437, 1319, 1294, 1285, 1276, 1230, 1219, 1203, 1163, 1116, 1029, 961, 907, 817, 792, 770, 731 cm^{-1}. ^{1}H NMR (500 MHz, DMSO-d6) δ 8.92 (s, 1H), 8.31 (s, 1H), 7.85 (d, J = 3.2 Hz, 1H), 7.67 (d, J = 3.5 Hz, 1H), 7.35 (d, J = 4.0 Hz, 1H), 7.30 (d, J = 4.0 Hz, 1H), 6.81 (d, J = 3.2 Hz, 1H). ^{13}C NMR (126 MHz, DMSO-d6) δ 161.5, 142.6, 140.1, 139.9, 134.8, 133.8, 133.5, 132.9, 132.8, 130.9, 130.8, 125.5, 125.0, 121.6, 115.5, 104.2, 52.4. LCMS (ESI): m/z calcd for $C_{11}H_7BrN_2S$ (M+H) 279.2 found: 281.2.

4b

General procedure of N-arylation reactions **G1** was applied starting with **1b** (73 mg, 0.62 mmol) and **2** (150 mg, 0.62 mmol). The residue was subjected to column chromatography using heptane to give **4b** (23 mg, 17 %) as white solid. IR (neat) 3087, 1674, 1611, 1557, 1496, 1477, 1462, 1417, 1362, 1206, 1179, 1080, 1010, 971, 924, 847, 777, 767, 732 cm^{-1}. ^{1}H NMR (500 MHz, DMSO-d6) δ 8.41 (d, J = 0.9 Hz, 1H), 7.94 – 7.89 (m, 2H), 7.58 (ddd, J = 8.5, 7.0, 1.1 Hz, 1H), 7.35 – 7.32 (m, 1H), 7.31 (d, J = 4.1 Hz, 1H), 7.28 (d, J = 4.1 Hz, 1H). ^{13}C NMR (126 MHz, DMSO-d6) δ 142.7, 138.0, 136.9, 129.8, 128.5, 125.0, 122.5, 121.8, 115.7, 110.6, 106.1. LCMS (ESI): m/z calcd for $C_{11}H_7BrN_2S$ (M+H) 279.2 found: 281.1.

4c

General procedure of N-arylation reactions **G1** was applied starting with **1c** (73 mg, 0.62 mmol) and **2** (150 mg, 0.62 mmol). The residue was subjected to column chromatography using [heptane/acetone (0→30 %)] to give **4c** (20 mg, 11 %) as white solid. IR (neat) 1597, 1551, 1490, 1405, 1343, 1285, 1269, 1235, 969, 798, 778, 767 cm^{-1}. ^{1}H NMR (500 MHz, DMSO-d6) δ 8.99 (s, 1H), 8.48 (dd, J = 4.8, 1.4 Hz, 1H), 8.24 (dd, J = 8.0, 1.4 Hz, 1H), 7.46 (d, J = 4.1 Hz, 1H), 7.44 (dd, J = 8.0, 4.8 Hz, 1H), 7.33 (d, J = 4.1 Hz, 1H). ^{13}C NMR (126 MHz, DMSO-d6) δ 145.2, 144.0, 140.6, 136.1, 135.3, 129.3, 128.8, 119.9, 119.6, 108.8. m/z calcd for $C_{10}H_6BrN_3S$ (M+H) 280.1 found: 282.1.

4d

General procedure of N-arylation reactions **G1** was applied starting with **1d** (73 mg, 0.62 mmol) and **2** (150 mg, 0.62 mmol). The residue was subjected to column chromatography using [heptane/acetone (0→20 %)] to give **4d** (62 mg, 36 %) as colorless oil. IR (neat) 1611, 1555, 1489, 1459, 1443, 1296, 1274, 1223, 1189, 1136, 968, 914, 793, 779, 762, 738 cm^{-1}. ^{1}H NMR (500 MHz, DMSO-d6) δ 8.53 (s, 1H), 7.80 – 7.76 (m, 1H), 7.62 – 7.59 (m, 1H), 7.39 – 7.30 (m, 4H). ^{13}C NMR (126 MHz, DMSO-d6) δ 144.5, 143.6, 137.3, 134.3, 130.4, 124.6, 123.9, 123.6, 120.5, 110.9, 109.5. LCMS (ESI): m/z calcd for $C_{11}H_7BrN_2S$ (M+H) 279.2 found: 281.2.

HS-276

General procedures of **G3** were applied starting with **4a** (31 mg, 0.26 mmol) and **5** (40 mg, 0.13 mmol), and **G2** starting with **1a** (27 mg, 0.97 mmol) and **3** (29 mg, 0.11 mmol). Column chromatography [CH_2Cl_2/MeOH (0→5 %)] afforded **HS-276** (23 mg, 70 % for **G3**, and 21 mg, 47 % for **G2**) as yellow solid. IR (neat) 1708, 1564, 1527, 1481, 1440, 1306, 1296, 1229, 1206, 1189, 1161, 1130, 1100, 1059, 866, 798, 783, 771, 740, 717 cm^{-1}. ^{1}H NMR (500 MHz, DMSO-d6) δ 9.04 (s, 1H), 7.95 – 7.94 (m, 1H), 7.80 (d, J = 4.0 Hz, 1H), 7.69 (dd, J = 5.4, 1.1 Hz, 1H), 7.59 (d, J = 4.0 Hz, 1H), 7.48 (dd, J = 5.1, 4.0 Hz, 2H), 6.86 (dd, J = 3.3, 0.8 Hz, 1H), 3.85 (s, 3H).^{13}C NMR (126 MHz, DMSO-d6) δ 161.5, 142.6, 140.1, 139.9, 134.9, 133.8, 133.5, 132.9, 130.9, 130.8, 125.5, 125.0, 121.6, 115.5, 104.2, 77.9, 52.4. LCMS (ESI): m/z calcd for $C_{17}H_{12}N_2O_2S_2$ (M+H) 340.4 found: 341.7.

HS-300

General procedures of **G3** were applied starting with **4b** (41 mg, 0.15 mmol) and **5** (47 mg, 0.18 mmol), and **G2** starting with **1b** (31 mg, 0.26 mmol) and **3** (40 mg, 0.13 mmol). Column chromatography [heptane/acetone (0→25 %)] yielded **HS-300** (22 mg, 43 % for **G3** and 35 mg, 78 % for **G2**) as yellow solid. IR (neat) 1704, 1567, 1489, 1450, 1422, 1350, 1321, 1276, 1250, 1194, 1092, 1063, 931, 802, 767, 743. ^{1}H NMR (500 MHz, $CDCl_3$) δ 8.20 (d, J = 0.9 Hz, 1H), 7.83 – 7.79 (m, 2H), 7.72 (d, J = 3.9 Hz, 1H), 7.55 – 7.50 (m, 1H), 7.31 – 7.27 (m, 1H), 7.24 (d, J = 4.0 Hz, 1H), 7.18 (d, J = 4.0 Hz, 1H), 7.15 (d, J

= 3.9 Hz, 1H), 3.91 (s, 3H). ^{13}C NMR (126 MHz, DMSO-d6) δ 143.0, 142.8, 138.0, 137.2, 134.9, 130.3, 129.0, 128.7, 125.8, 125.3, 124.6, 122.7, 122.0, 115.3, 111.0, 52.4. LCMS (ESI): m/z calcd for $C_{17}H_{12}N_2O_2S_2$ (M+H) 340.4 found: 341.5.

HS-302

General procedures of **G3** were applied starting with **4c** (60 mg, 0.21 mmol) and **5** (69 mg, 0.26 mmol), and **G2** starting with **1c** (47 mg, 0.40 mmol) and **3** (60 mg, 0.2 mmol). Column chromatography [CH_2Cl_2/MeOH (0→5 %)] yielded **HS-302** (33 mg, 45 % for **G3**, and 11 mg, 16 % for **G2**) as off white solid. IR (neat) 1697, 1600, 1556, 1527, 1494, 1470, 1446, 1413, 1289, 1236, 1256, 1192, 1140, 1096, 781, 767, 743. ^1H NMR (500 MHz, $CDCl_3$) δ 8.52 (dd, J = 4.8, 1.5 Hz, 1H), 8.34 (d, J = 4.3 Hz, 1H), 8.16 (dd, J = 8.1, 1.5 Hz, 1H), 7.72 (d, J = 3.9 Hz, 1H), 7.36 (dd, J = 8.1, 4.7 Hz, 1H), 7.32 (d, J = 3.9 Hz, 1H), 7.25 (d, J = 4.0 Hz, 1H), 7.19 (d, J = 3.9 Hz, 1H), 3.91 (s, 3H). ^{13}C NMR (126 MHz, $CDCl_3$) δ 162.5, 146.6, 145.6, 143.2, 142.7, 135.7, 135.6, 134.4, 133.6, 132.2, 128.9, 124.4, 123.9, 120.5, 119.7, 52.4. LCMS (ESI): m/z calcd for $C_{16}H_{11}N_3O_2S_2$ (M+H) 341.4 found: 342.6.

HS-315

General procedures of **G3** were applied starting with **4d** (41 mg, 0.15 mmol) and **5** (47 mg, 0.18 mmol), and **G2** starting with **1d** (31 mg, 0.26 mmol) and **3** (40 mg, 0.13 mmol). Column chromatography [CH_2Cl_2/MeOH (0→5 %)] yielded **HS-315** (30 mg, 60 % for **G3**, and 5 mg, 11 % for **G2**) as yellow solid. IR (neat) 1709, 1568, 1483, 1447, 1439, 1293, 1185, 1109, 1100, 1054, 921, 869, 792, 779, 761, 741, 732 cm^{-1}. ^1H NMR (500 MHz, DMSO-d6) 8.08 (s, 1H), 7.86 (ddd, J = 6.0, 2.1, 0.6 Hz, 1H), 7.72 (d, J = 3.9 Hz, 1H), 7.61 (ddd, J = 3.8, 2.1, 0.6 Hz, 1H), 7.40 – 7.34 (m, 2H), 7.25 (d, J = 1.6 Hz, 1H), 7.18 (d, J = 3.9 Hz, 1H), 7.11 (d, J = 3.9 Hz, 1H), 3.90 (s, 3H). ^{13}C NMR (126 MHz, DMSO-d6) δ 162.4, 143.8, 142.9, 142.9, 137.1, 134.5, 134.4, 134.2, 132.4, 124.6, 124.5, 124.2, 123.6, 122.6, 121.0, 110.6, 52.5. LCMS (ESI): m/z calcd for $C_{17}H_{12}N_2O_2S_2$ (M+H) 340.4 found: 341.5.

HS-277

General procedure of hydrolysis **G4** was performed starting with **HS-276** (15 mg, 0.044 mmol). The product **HS-277** was obtained as yellow solid. Yield as free acid (12 mg, 84 %).

IR (neat) 1562, 1486, 1442, 1394, 1332, 1163, 1031, 804, 768. ^{1}H NMR (500 MHz, DMSO-d6) δ 8.99 (s, 1H), 8.29 (d, J = 5.3 Hz, 1H), 7.91 (d, J = 3.2 Hz, 1H), 7.68 (dd, J = 5.4, 1.1 Hz, 1H), 7.37 (d, J = 3.9 Hz, 1H), 7.30 (d, J = 3.9 Hz, 1H), 7.19 (dd, J = 10.0, 3.6 Hz, 2H), 6.82 (dd, J = 3.2, 0.8 Hz, 1H). ^{13}C NMR (126 MHz, DMSO-d6) δ 164.3, 147.9, 139.9, 137.5, 136.3, 133.8, 133.6, 133.5, 133.1, 133.1, 128.0, 124.0, 122.6, 121.8, 115.5, 103.8. LCMS (ESI): m/z calcd for $C_{16}H_{10}N_2O_2S_2$ (M+H) 326.4 found: 327.6.

HS-301

General procedure of hydrolysis **G4** was performed starting with **HS-300** (20 mg, 0.059 mmol). The product was obtained as yellow solid. Yield as free acid (18 mg, 93 %).

IR (neat) 1556, 1489, 1450, 1392, 1368, 935, 803, 767, 732 cm^{-1}. ^{1}H NMR (500 MHz, DMSO-d6) δ 8.42 (d, J = 0.9 Hz, 1H), 7.98 (dd, J = 8.6, 0.8 Hz, 1H), 7.93 (dt, J = 8.1, 0.9 Hz, 1H), 7.59 (ddd, J = 8.3, 6.9, 1.1 Hz, 1H), 7.39 (d, J = 4.0 Hz, 1H), 7.36 – 7.31 (m, 1H), 7.25 (d, J = 4.0 Hz, 1H), 7.16 (s, 2H). δ ^{13}C NMR (126 MHz, DMSO-d6) 164.0, 147.8, 140.3, 138.1, 136.6, 136.5, 132.0, 128.4, 127.8, 125.0, 123.6, 122.6, 122.4, 121.8, 115.8, 110.8. LCMS (ESI): m/z calcd for $C_{16}H_{10}N_2O_2S_2$ (M+H) 326.4 found: 327.7.

HS-303

General procedure of hydrolysis **G4** was performed starting with **HS-302** (20 mg, 0.059 mmol). The product was obtained as off white solid. Yield as free acid (8 mg, 83 %).

IR (neat)1567, 1498, 1433, 1399, 1363, 1292, 1273, 1241, 1063, 880, 804, 767. ^{1}H NMR (500 MHz, DMSO-d6) δ 9.08 (s, 1H), 8.53 (dd, J = 4.8, 1.4 Hz, 1H), 8.26 (dd, J = 8.0, 1.4 Hz, 1H), 7.69 (d, J = 3.9 Hz, 1H), 7.65 (d, J = 4.1 Hz, 1H), 7.54 (d, J = 4.0 Hz, 1H), 7.48 – 7.43 (m, 2H). ^{13}C NMR (126 MHz, DMSO-d6) δ 162.6, 145.3, 144.8, 143.7, 135.3, 135.0, 134.1, 131.6, 128.4, 124.7, 124.2, 119.5, 119.2. LCMS (ESI): m/z calcd for $C_{15}H_9N_3O_2S_2$ (M+H) 327.4 found: 328.4

HS-316

General procedure of hydrolysis **G4** was performed starting with **HS-315** (20 mg, 0.059 mmol). The product was obtained as yellow solid. Yield as free acid (16 mg, 83 %).

IR (neat) 1563, 1494, 1443, 1380, 1298, 1233, 774, 736. ^{1}H NMR (500 MHz, DMSO-d6) δ 8.59 (s, 1H), 7.81 – 7.78 (m, 1H), 7.72 – 7.69 (m, 1H), 7.41 – 7.33 (m, 4H), 7.22 (d, J = 3.6 Hz, 1H), 7.20 (d, J = 3.7 Hz, 1H). ^{13}C NMR (126 MHz, DMSO-d6) δ 164.0, 148.1, 144.0, 143.3, 136.2, 134.8, 134.5, 133.8, 128.1, 124.3, 124.1, 123.1, 122.9, 122.6, 120.1, 110.7.

LCMS (ESI): m/z calcd for $C_{16}H_{10}N_2O_2S_2$ (M+H) 326.4 found: 327.6

Optical characterization of ligands

Stock solutions (1.5 mM) of ligands were prepared by solubilizing powder of b-TVBT2, HS-276, HS-277, HS-300, HS-302, HS-315 and HS-316 in DMSO, HS-303 in 50% DMSO, and HS-301 in dH_2O. For absorbance measurements, the stock solutions were further diluted to 50 μM in DMSO, 50% DMSO (HS-303) or dH_2O (HS-301). For fluorescence analysis, the stock solutions were further diluted to 600 nM in DMSO, 50% DMSO (HS-303) or dH_2O (HS-301) or in phosphate buffered saline (PBS, 10 mM phosphate, 140 mM NaCl, 2.7 mM KCl, pH 7.4). The absorbance and emission spectrum of each ligand was collected on a Tecan Infinite M1000 Pro plate reader (Tecan) using a quartz microplate (Hellma) to reduce background absorbance in the UV range. When collecting the emission spectrum, excitation wavelength 340 nm was used for HS-303 and HS-316, 360 nm for HS-276, HS-277, HS-301, HS-302, HS-315, and 380 nm for HS-300.

Human tissue

Frozen brain tissue from a neuropathologically confirmed case of Alzheimer's disease (AD) was obtained from the Dementia Laboratory at the Department of Pathology and Laboratory Medicine, Indiana University School of Medicine, Indianapolis, USA. The studies carried out at the Indiana University School of Medicine were reviewed and approved by the Indiana University Institutional Review Board and informed consent was obtained from the patient or their next of kin. The

experiments performed at Linköping University were reviewed and approved by a national ethical committee (approval number 2020-01197).

Ligand staining and spectral analysis

Frozen brain tissue from a neuropathologically confirmed case of AD was obtained from the Dementia Laboratory at the Department of Pathology and Laboratory Medicine, Indiana University School of Medicine, Indianapolis, USA. For ligand staining, sections of the brain tissue (frontal cortex, 10 µm) were fixed in 99.7% EtOH for 10 min. The sections were rehydrated in 50% EtOH for 2 min, dH_2O for 2x2 min and PBS for 10 min. Ligand HS-276, HS-277, HS-300, HS-301, HS-302, HS-303, HS-315 or HS-316 were diluted to 100 nM in PBS and added to the sections. After 30 min incubation at RT, the sections were washed with PBS three times and then mounted with Dako mounting medium for fluorescence (Agilent). The mounting medium was allowed to solidify for at least 24 h before the result was analysed. The emission spectrum of each ligand when binding to Aβ plaques or NFTs (HS-300 only) in AD brain tissue was collected using an inverted Zeiss LSM 780 laser scanning confocal microscope (Zeiss) exciting the ligands at 405 nm. For each ligand, at least 29 Aβ plaques were included in the analysis, and for each Aβ plaque, the emission spectrum for 1-7 regions was collected. In the section stained with HS-300, seven NFTs were analysed including 3-8 regions for each deposit. To detect cerebral amyloid angiopathy lesions, a frozen AD brain tissue section was fixed in ethanol and stained with 600 nM of LCO ligand HS-84 as described above.

Ligand double staining

Frozen brain sections (frontal cortex, 10 µm) from AD patient were fixed in 99.7% EtOH and then rehydrated as described above. For ligand double staining, tissue sections were incubated in PBS buffer containing 100 nM b-TVBT2 in combination with 100 nM HS-276 or 100 nM HS-300 for 30 min at RT. The sections were washed in PBS three times and then mounted (Agilent). Fluorescence images were collected from the HS-300 and b-TVBT2 double-labelled sample using an inverted Zeiss LSM 780 laser scanning confocal microscope (Zeiss) exciting HS-300 at 405 nm and b-TVBT2 at 565 nm.

Autofluorescence from lipofuscin granules was visualized by exciting the sample at 490 nm. Fluorescence lifetime imaging microscopy (FLIM) was performed on the HS-276 and b-TVBT2 double-labelled section using an inverted Zeiss LSM 780 laser scanning confocal microscope (Zeiss) equipped with a FLIM module (see FLIM section below for details). In the FLIM analysis, ligands were excited at 405 nm (HS-276) and 565 nm (b-TVBT2).

Fluorescence lifetime imaging microscopy (FLIM)

Fluorescence lifetime imaging microscopy was performed using an inverted Zeiss LSM 780 laser scanning confocal microscope (Zeiss) equipped with a 32 channel QUASAR GaAsP spectral array detector. After being guided through the direct coupling confocal port of the Zeiss LSM 780 scanning unit, the emitted photons were detected by a Becker & Hickl HPM-100-40 hybrid photomultiplier tube (PMT) (Becker & Hickl GmbH). Data were recorded by a Simple-Tau 152 system (SPC-150 TCSPC FLIM module) with the instrument recording software SPCM version 9.42 in the FIFO image mode using 256 time-channels. A Plan-Apochromat 40x/1.3 Oil DIC M27 objective lens was used, and the pinhole set to 43 µm. For excitation at 405 nm, a diode laser pulsed at 50 MHz was used, and for excitation at 565 nm, a pulsed In Tune laser with a repetition rate of 40 MHz was applied. Data were analyzed using FLIM data analysis software SPCImage version 3.9.4 (Becker & Hickl GmbH).

Ligand and antibody double staining

To accomplish double labelling of Aβ plaques or neurofibrillary tangles with ligand and antibody, frozen brain sections (frontal cortex, 10 µm) from AD patient were fixed in pre-cooled acetone for 5 min at -20°C. The sections were allowed to dry for 30 min at RT before being incubated in PBS for 1 min (to remove OCT embedding compound) and then in PBS with 5% normal goat serum (blocking buffer) for 30 min at RT. Anti-Aβ-antibody 6E10 (BioLegend) or anti-phospho-tau-antibody AT8 (Thermo Scientific) were diluted 1:1000 and 1:500, respectively, in blocking buffer and added to the sections. After 2h at RT, the sections were washed in PBS for 3x5 min and then incubated for 1h at RT with goat anti-mouse secondary antibody conjugated to Alexa 594 or Alexa 647 (Thermo Scientific)

diluted 1:400 in blocking buffer. The sections were washed in PBS for 3x5 min and 100 nM of ligand b-TVBT2, HS-276, HS-277, HS-300, HS-301, HS-302, HS-303, HS-315 or HS-316 diluted in PBS, was added. To investigate autofluorescence from Aβ plaques, one section was incubated with PBS only. After 30 min at RT, excess ligand was removed by washing in PBS and the sections were mounted (Agilent). The mounting medium was allowed to solidify for at least 24 h before the result was analysed using an inverted Zeiss LSM 780 laser scanning confocal microscope (Zeiss) using excitation wavelengths 405 nm (all ligands except b-TVBT2), 561 nm (b-TVBT2), 595 nm (Alexa 594), 633 nm (Alexa 647), 458 nm (lipofuscin) or 490 nm (lipofuscin).

Ligand binding study

To explore the binding mode of the new ligand scaffold, frozen brain tissue sections (10 μm, frontal cortex) from AD patient were fixed using 99.7% EtOH and then rehydrated as described above. Five sections were included in total and all steps were performed at RT. Section one and two were incubated with 100 nM HS-276 or 10 μM HS-169 for 30 min, washed three times in PBS and then mounted. Section three and four were incubated with 10 μM q-FTAA-CN or HS-169 for 30 min, washed three times in PBS and then stained with 100 nM HS-276 for 30 min. The sections were washed in PBS three times before being mounted. Section five was incubated with 10 μM HS-169 for 30 min, washed three times in PBS and then labelled with 100 nM q-FTAA-CN for 30 min. After washing in PBS three times, the section was mounted. For all sections, Dako mounting medium for fluorescence was used (Agilent). To investigate ligand binding FLIM analysis was performed as described above. The samples were excited at 405 nm.

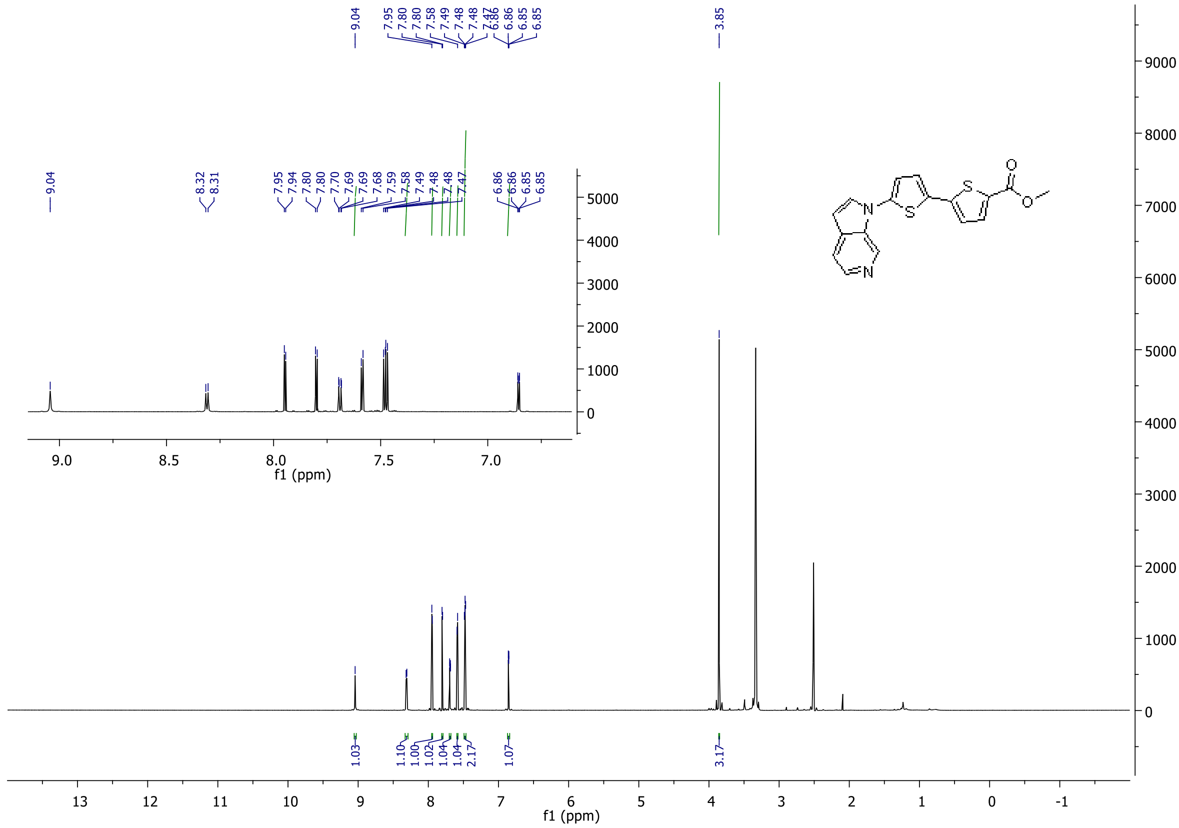
9.04
7.95
7.94
7.80
7.80
7.58
7.49
7.48
7.48
7.47
6.86
6.86
6.85
6.85
3.85
9.04
8.32
8.31
7.70
7.69
7.69
7.68
7.59
7.58
1.03
1.10
1.00
1.02
1.04
1.04
2.17
1.07
3.17
f1 (ppm)

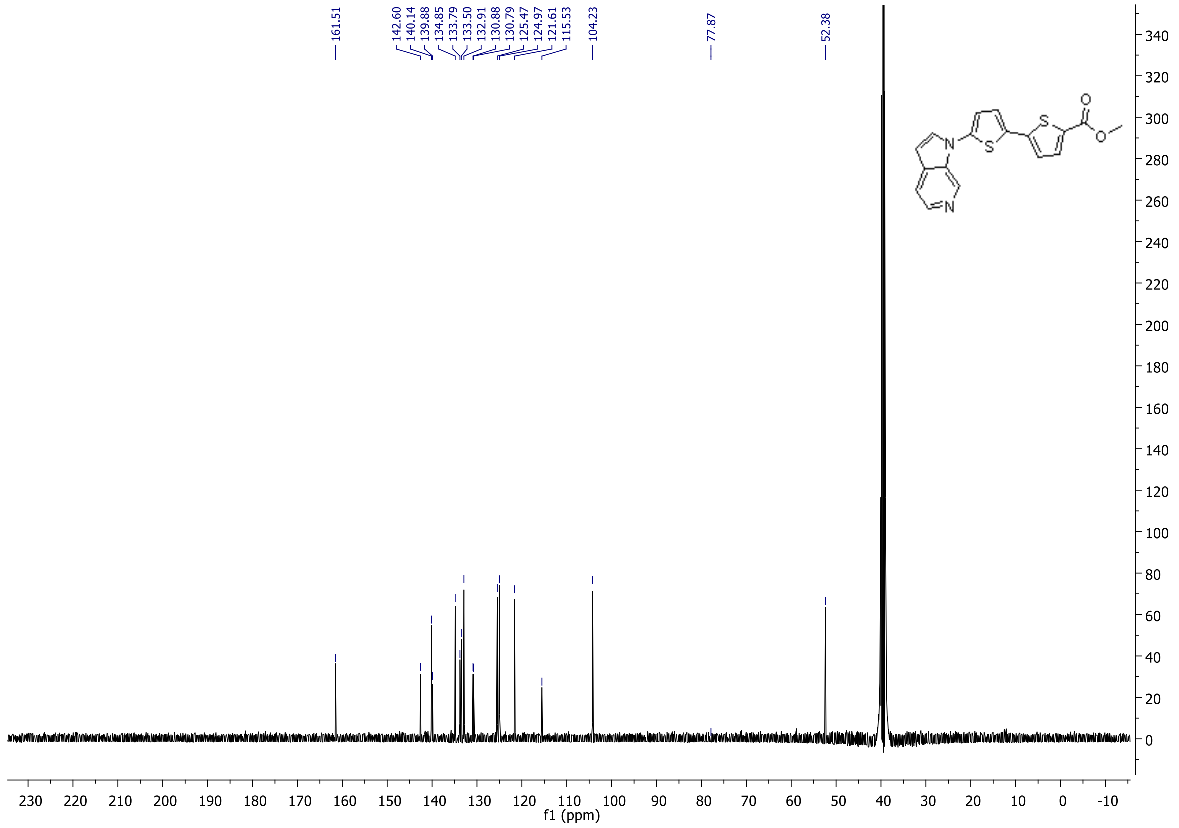
161.51
142.60
140.14
139.88
134.85
133.79
133.50
132.91
130.88
130.79
125.47
124.97
121.61
115.53
104.23
77.87
52.38
f1 (ppm)

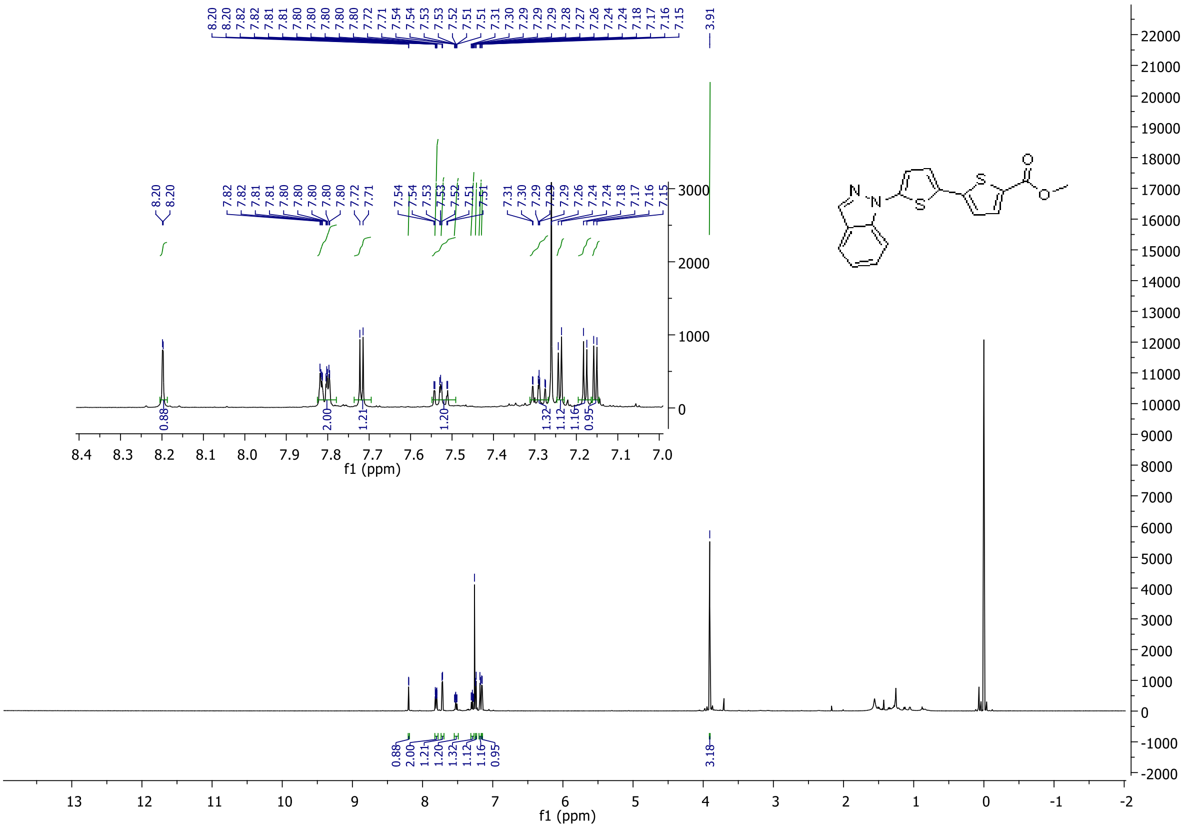

f1 (ppm)
f1 (ppm)

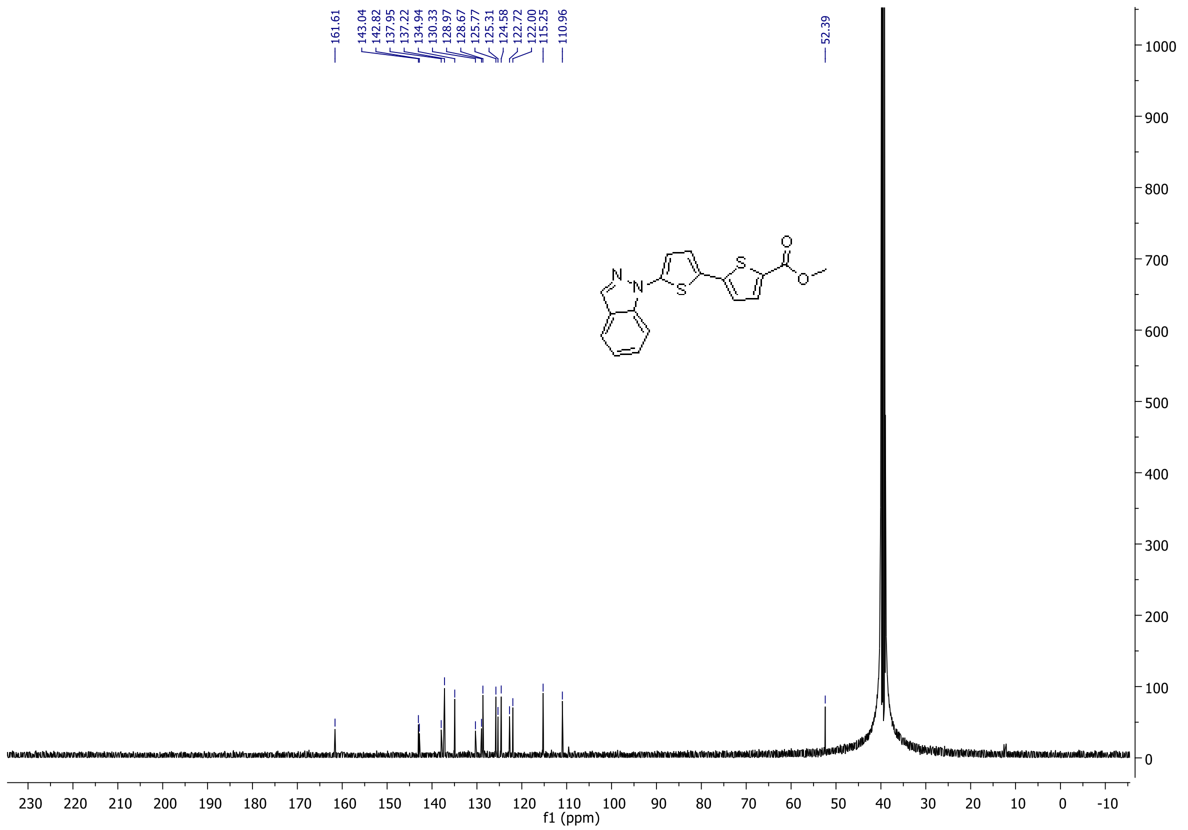
161.61
143.04
142.82
137.95
137.22
134.94
130.33
128.97
128.67
125.77
125.31
124.58
122.72
122.00
115.25
110.96
52.39
f1 (ppm)

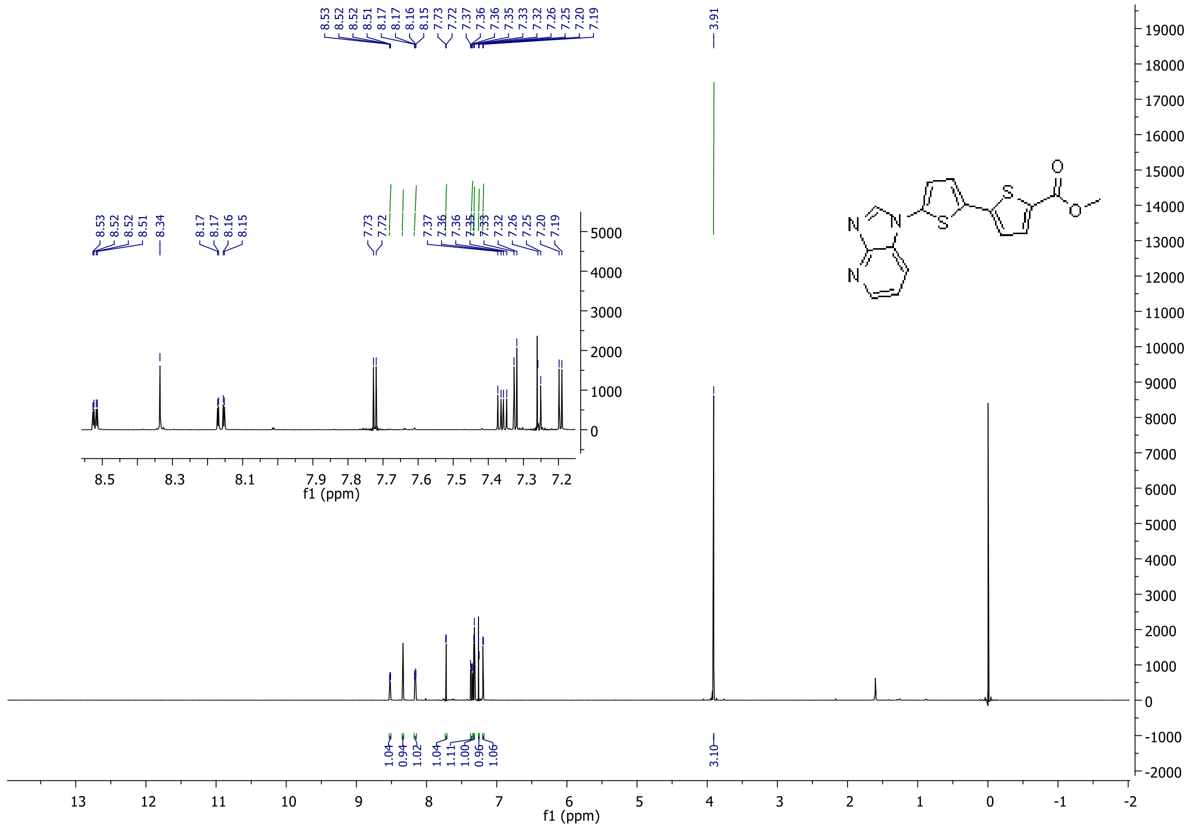
8.53
8.52
8.52
8.51
8.17
8.17
8.16
8.15
7.73
7.72
7.37
7.36
7.35
7.33
7.32
7.26
7.25
7.20
7.19
3.91
1.04
0.94
1.02
1.04
1.11
1.00
0.96
1.06
3.10
f1 (ppm)

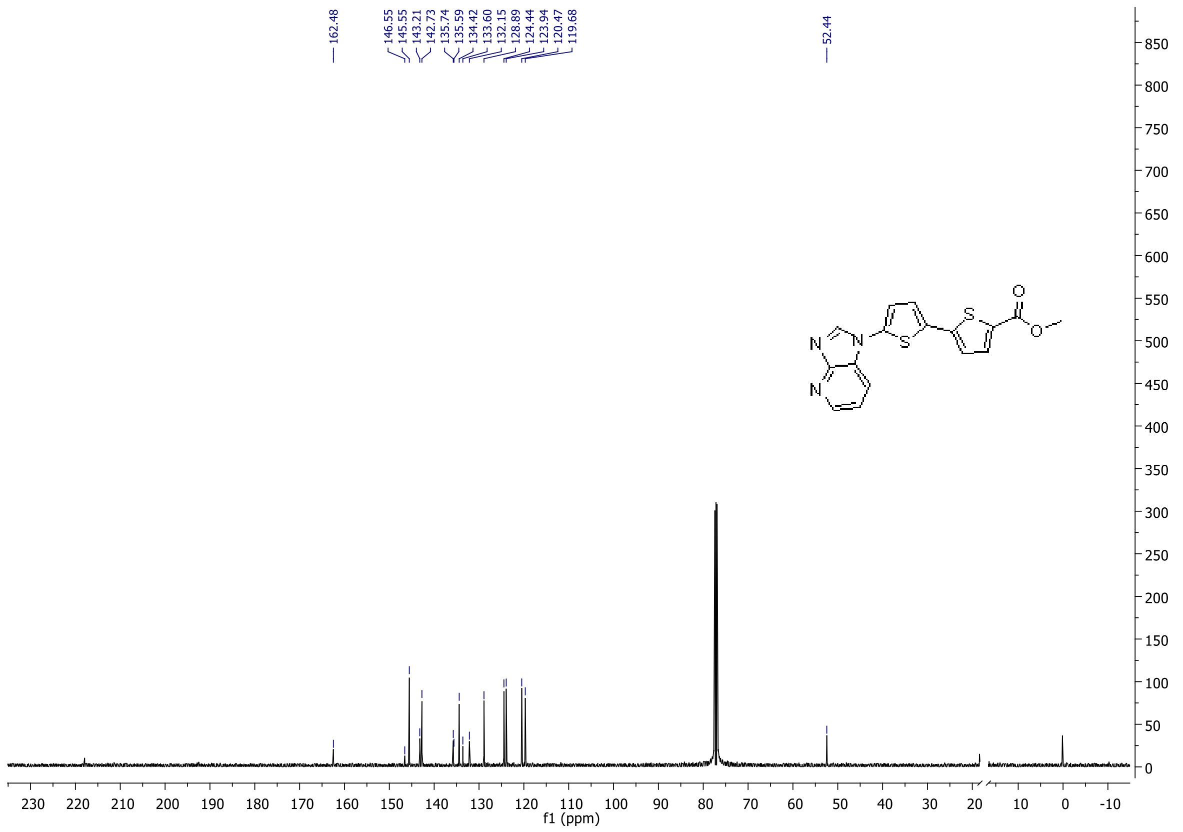

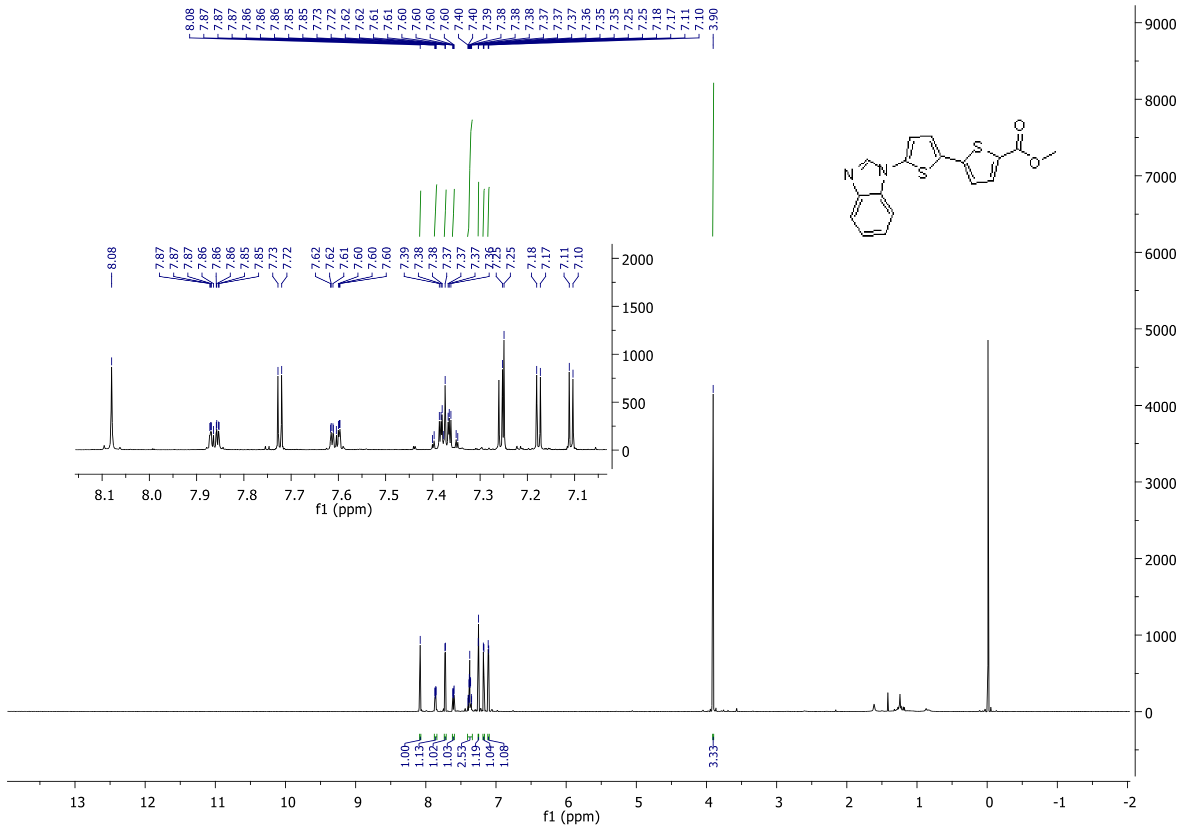

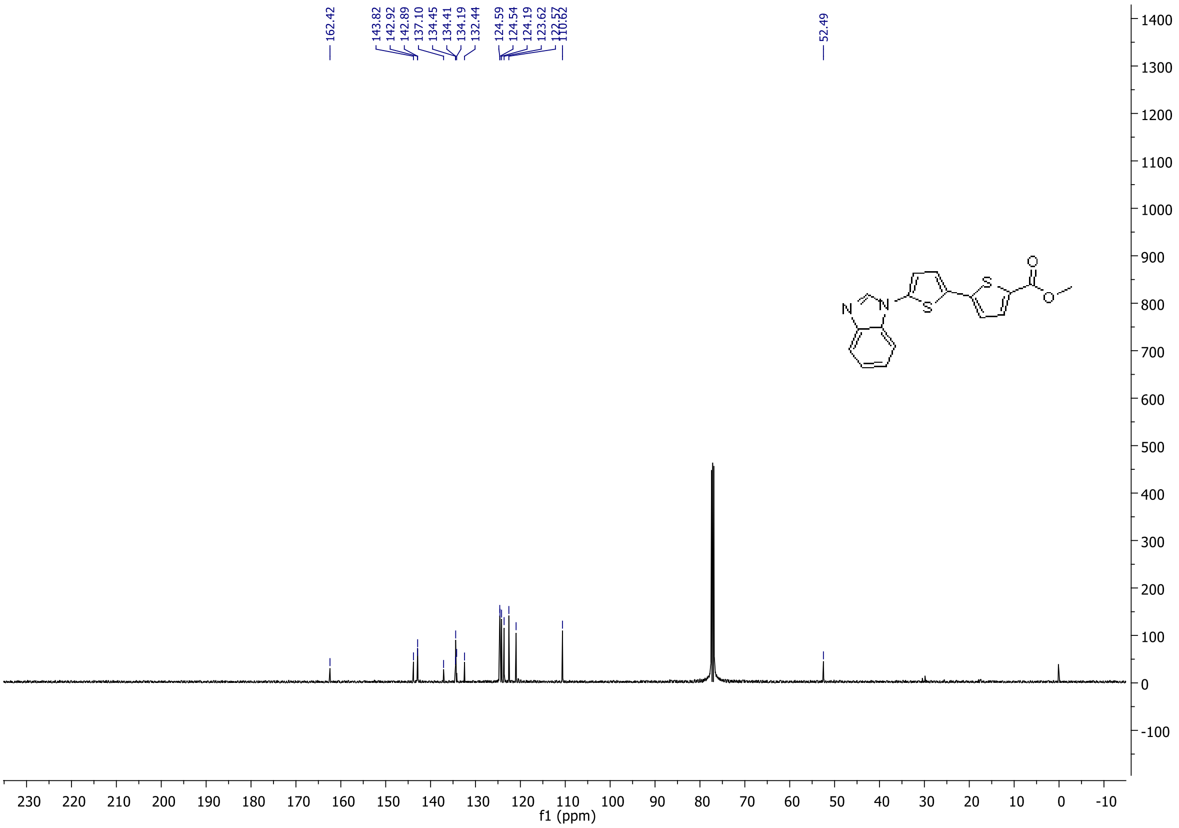
162.42
143.82
142.92
142.89
137.10
134.45
134.41
134.19
132.44
124.59
124.54
124.19
123.62
122.57
110.62
52.49
f1 (ppm)

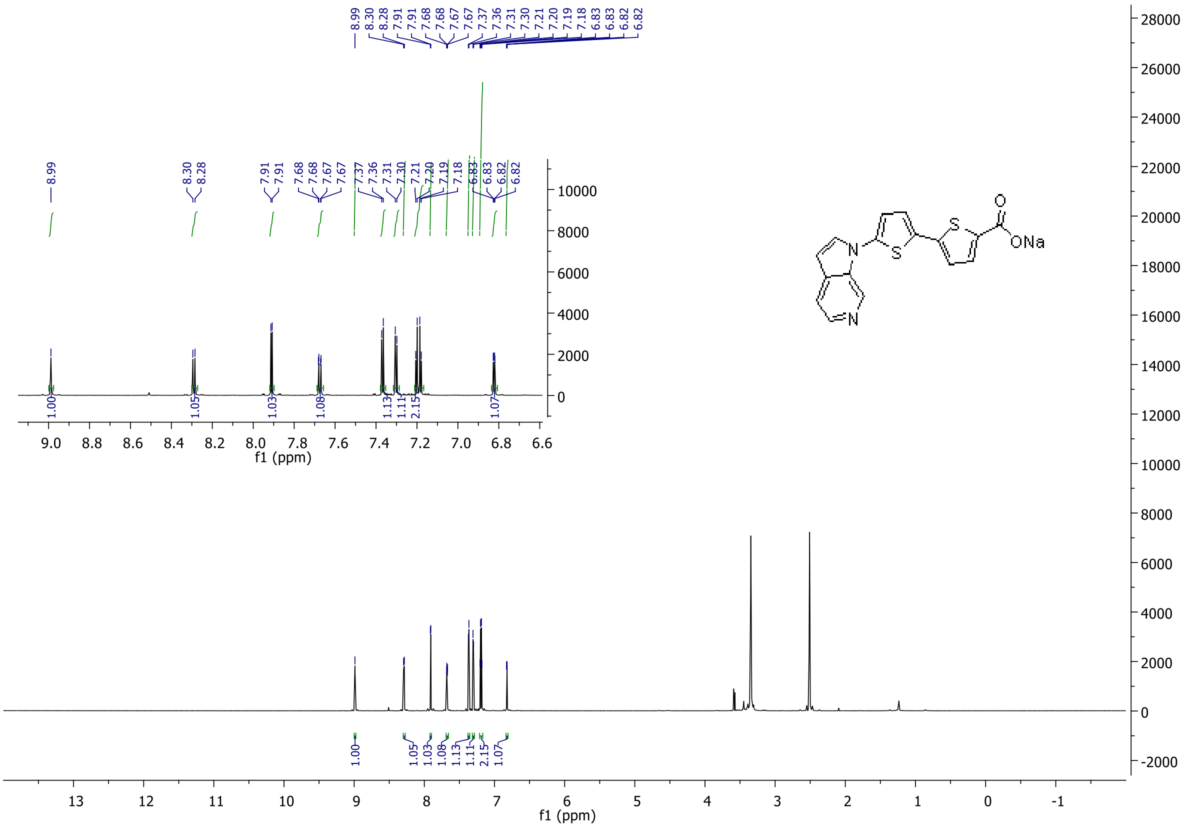
ONa
f1 (ppm)

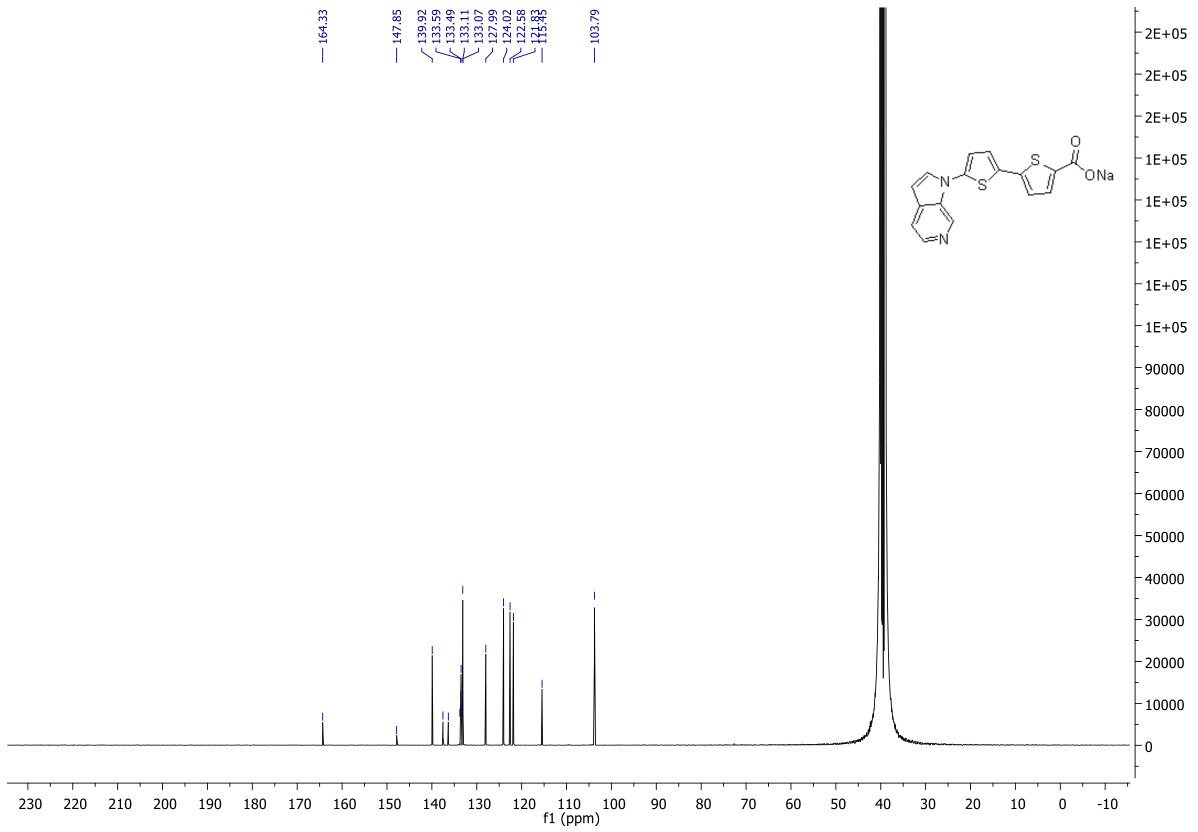

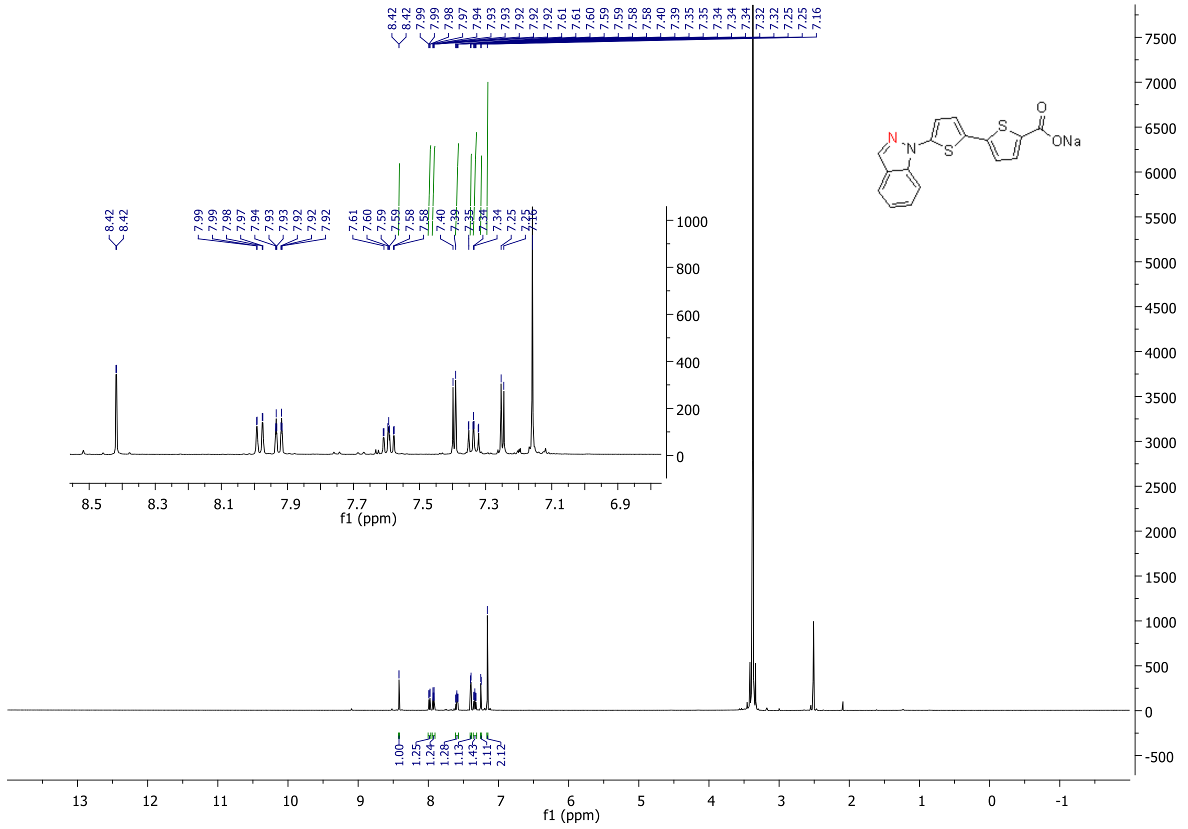

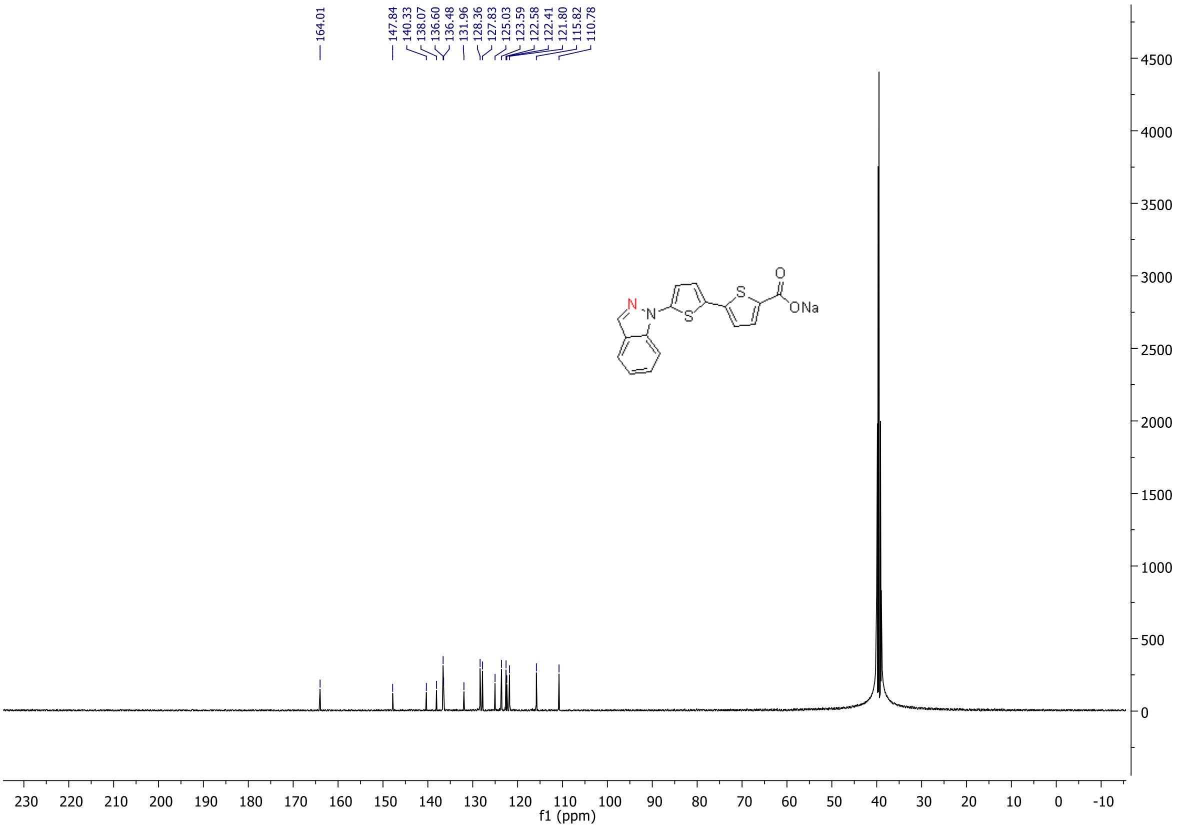
164.01
147.84
140.33
138.07
136.60
136.48
131.96
128.36
127.83
125.03
123.59
122.58
122.41
121.80
115.82
110.78
N
N
S
S
O
ONa
f1 (ppm)

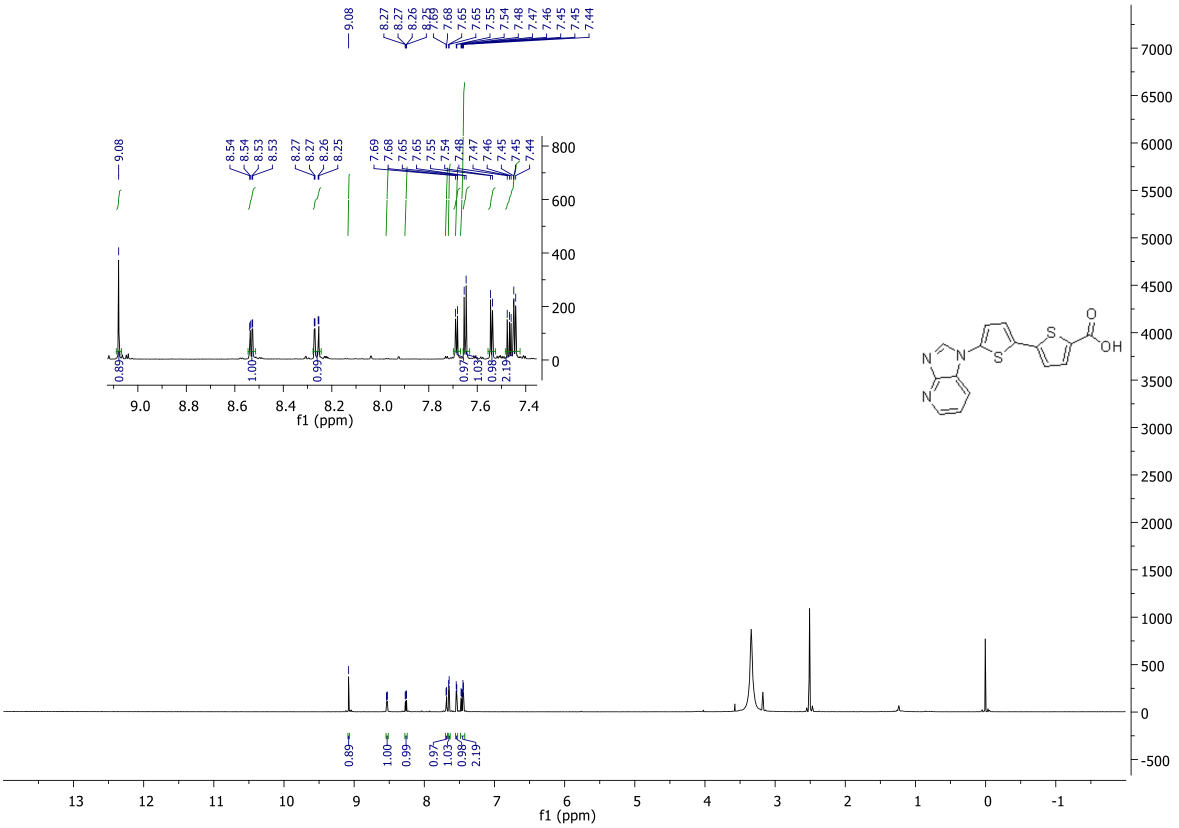
9.08
8.27
8.27
8.26
8.25
7.69
7.68
7.65
7.65
7.55
7.54
7.48
7.47
7.46
7.45
7.45
7.44
8.54
8.54
8.53
8.53
0.89
1.00
0.99
0.97
1.03
0.98
2.19
f1 (ppm)
N
N
N
S
S
O
OH

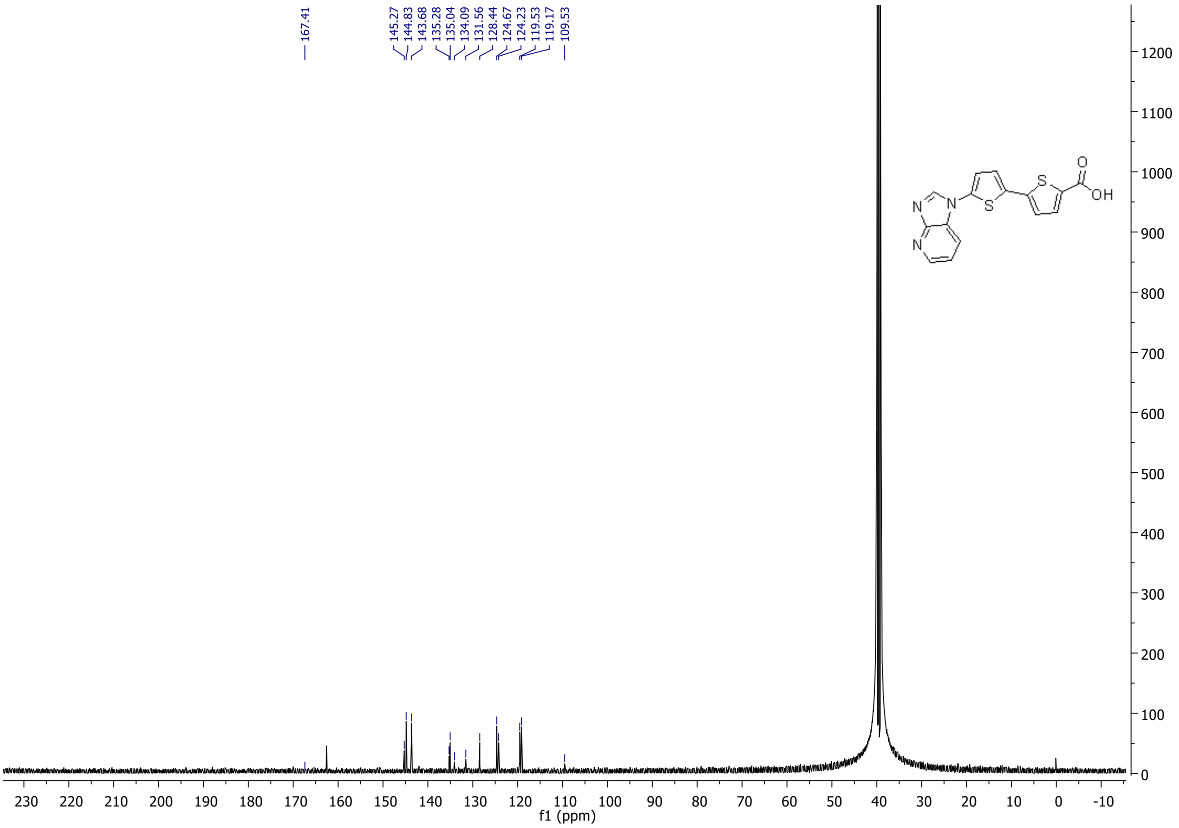
167.41
145.27
144.83
143.68
135.28
135.04
134.09
131.56
128.44
124.67
124.23
119.53
119.17
109.53
f1 (ppm)

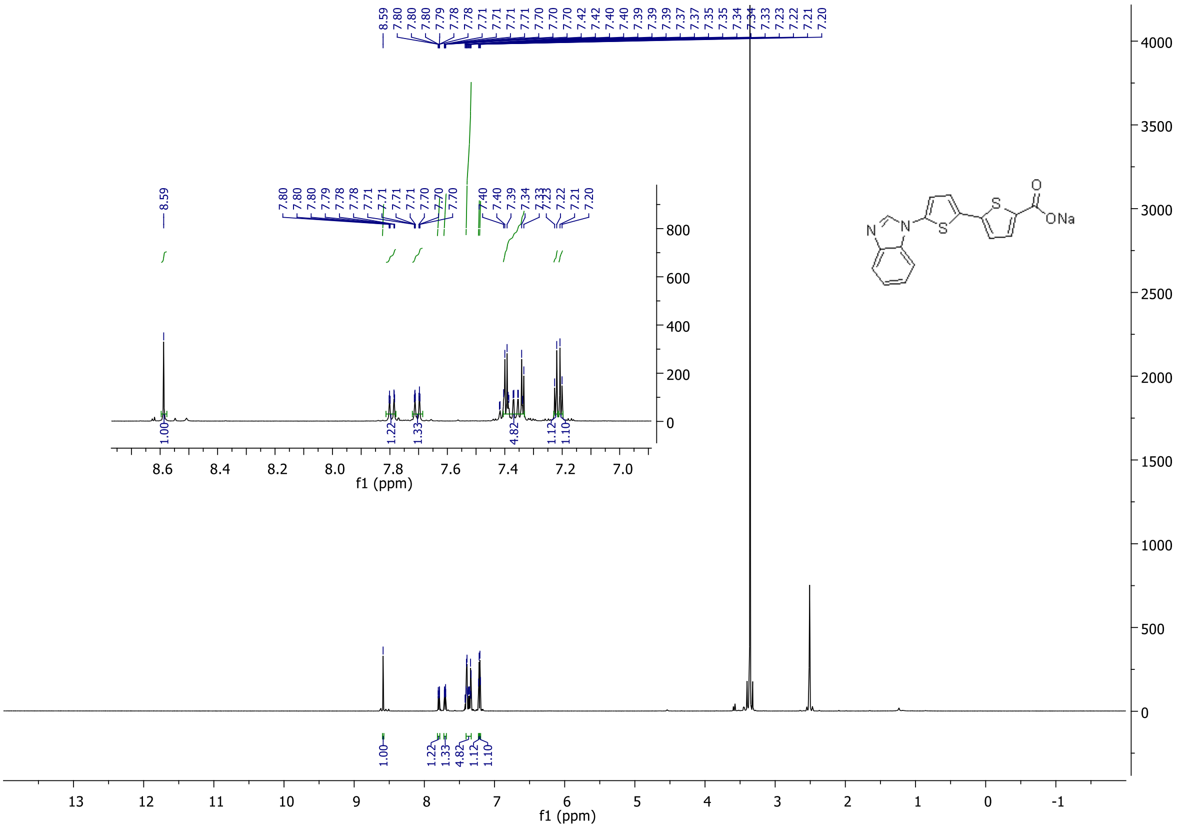
8.59
7.80
7.80
7.79
7.78
7.78
7.71
7.71
7.71
7.70
7.70
7.40
7.40
7.39
7.34
7.33
7.23
7.22
7.21
7.20
1.00
1.22
1.33
4.82
1.12
1.10
f1 (ppm)

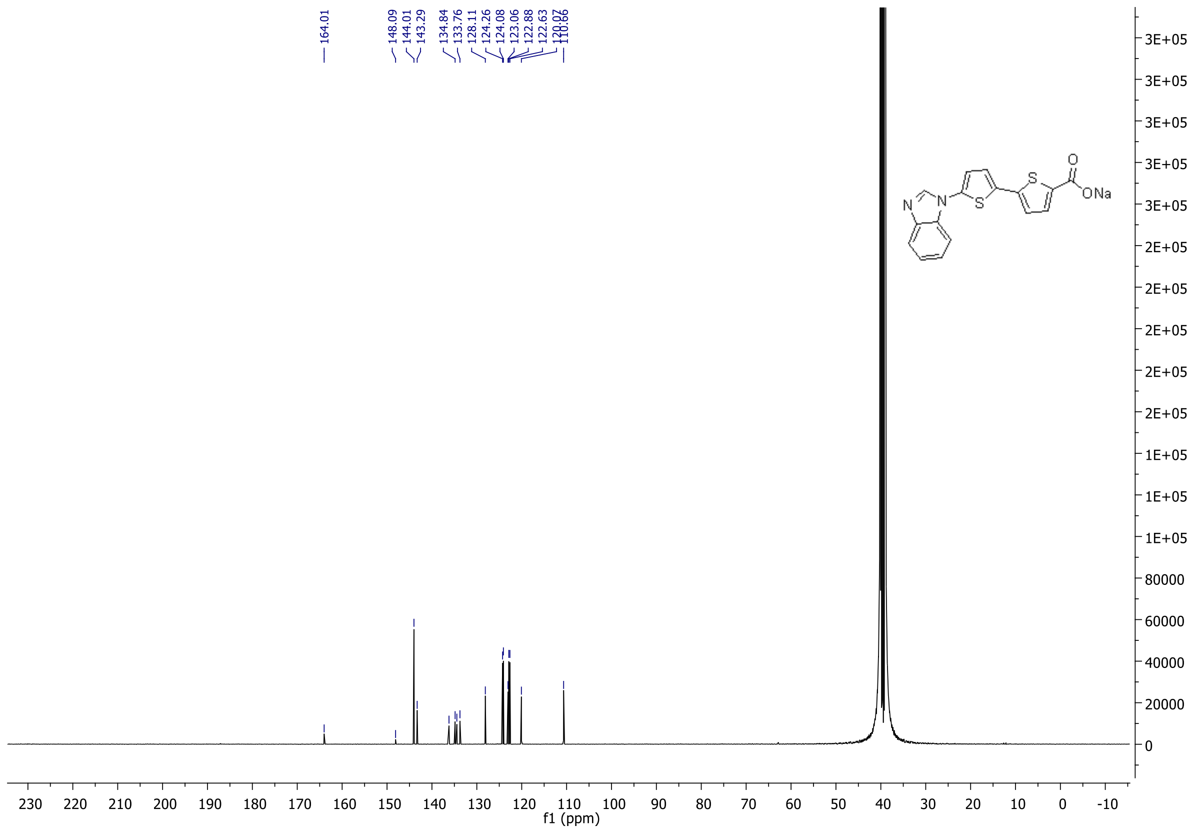
164.01
148.09
144.01
143.29
134.84
133.76
128.11
124.26
124.08
123.06
122.88
122.63
120.07
110.86
f1 (ppm)
ONa
